# A review on ethnobotany, phytochemistry, and pharmacology of the genus *Duhaldea* DC

**DOI:** 10.3389/fphar.2024.1479963

**Published:** 2024-11-15

**Authors:** Liangyin Shu, Kailin Li, Suyu Yang, Mengdie Hu, Xinrong Ming, Bingyuan Yan, Liangjun Guan, Shunli Xiao

**Affiliations:** ^1^ School of Pharmaceutical Sciences, Hunan University of Medicine, Huaihua, China; ^2^ School of Pharmacy, Shandong Second Medical University, Weifang, China; ^3^ School of Pharmacy, Baotou Medicai College, Baotou, China

**Keywords:** *Duhaldea* DC., ethnobotany, pharmacology, phytochemistry, traditional uses

## Abstract

The botanical drugs of genus *Duhaldea* DC. have been traditionally utilized in folk medicine for the treatment of a wide array of illnesses, encompassing fractures, bone wounds, carbuncles and poisoning, bronchitis, bruises, giddy with hypertension of qi, and lung deficiency cough. The genus *Duhaldea* DC. comprises 15 species widespread in Central, East, and Southeast Asia and 7 species (2 endemic) in China. The review aims to provide a systematic overview of ethnopharmacology, phytochemistry, and pharmacology of *Duhaldea* DC. and to explore the future therapeutic potential and scientific potential of this genus. The data were systematically collected from books and scientific databases such as PubMed, Web of Science, Google Scholar, CNKI, and doctoral and master’s theses. To date, a total of 352 metabolites have been isolated from this genus, and terpenoids, flavonoids, phenylpropanoids, and inositol angelates are the primary contributors to the pharmacological activities of *Duhaldea* DC. The crude extracts and isolated phytochemical metabolites from this genus have been shown to exhibit various pharmacological activities, including anti-inflammatory, antimicrobial, anti-osteoporotic, anticancer, and antioxidant activities. Despite notable advancements in our understanding of the chemical constituents and pharmacological properties of *Duhaldea* DC., it is absolutely crucial to conduct additional research into the pharmacology and toxicology of these species to definitively ascertain their safety, efficacy, and quality.

## 1 Introduction

Previously, plants of the genus *Duhaldea DC*. were considered to belong to the genus *Inula* L. (Cheng et al., 2014a; Li, 2017). However, in the last decade or so, plant taxonomists have concluded that these plants should become a separate genus under the family Asteraceae, and the scientific name of the genus has been revised to *Duhaldea* DC (Wu and Peter, 2011). *Duhaldea* DC consists of 15 species, mainly shrubs or perennial botanical drugs. The genus is widely distributed in Asia, with seven species in China, of which two are endemic (Wu and Peter, 2011). The stem is erect. The leaves are alternate and densely hairy. The capitulum is often solitary or found in dense terminal corymbs. It has multiple total bracts, with white edge flowers. The disk florets are bisexual, either yellow or whitish. The achenes are ellipsoid and hairy, with an epidermis featuring elongated crystals (Ding and Jin, 2017).

Species in the genus *Duhaldea* DC. are traditionally used for the treatment of stomachache, relieving rheumatism, fractures, bone wounds, asthma, headache, rheumatism, peptic ulcers, loose motions, indigestion, bronchitis, angeitides, vasculitis, and dizziness (Long, 2004; Yan et al., 2011; Jyoti et al., 2017; Huang et al., 2021a). *Duhaldea* DC. species are rich in sesquiterpene lactones, sesquiterpenes, phenylpropanoids, inositol derivatives, triterpenes, and flavonoids, which authenticate their medicinal importance (Baruah et al., 1982; Yan et al., 2010; Cheng et al., 2014b; Wu et al., 2015; Zheng et al., 2015). Anti-inflammatory, piscicidal, anti-osteoporotic, and anticancer activities exhibited by various extracts and metabolites isolated from the genus *Duhaldea* DC. showed its pharmacological importance (Yoshida et al., 1995; Xie et al., 2007; Wang et al., 2013; Tai et al., 2014). *D. nervosa* and *D. cappa* are the most popular, widely utilized, and highly investigated species in the genus despite the relatively low number of other species within the genus, which are more or less underexplored. This species is an important medicinal species extensively used in Asian countries to treat stomachache and rheumatism (Yan et al., 2011).

This review aims to establish a relationship between traditional uses and scientific studies by critically assessing the available literature on ethnopharmacology, phytochemistry, and pharmacology, possible mechanisms of action, and toxicology of the plant species from the genus *Duhaldea* DC. Furthermore, this review also highlights the various research gaps for the better exploitation of this genus and provides a baseline for future research studies. Related scientific literature studies up to March 2024 were collected from the following databases: PubMed, Elsevier, Web of Science, Springer, ScienceDirect, Wiley, ACS, CNKI, and doctoral and master’s theses. The search terms included “Duhaldea,” “rubricaulis,” “wissmanniana,” “lachnocephala,” “forrestii,” “nervosa,” “cappa,” “pterocaula,” “eupatorioides,” “cuspidata,” “griffithii,” “latifolia,” “revoluta,” “simonsii,” “xiaoheiyao,” “caoweiling,” and “baimianfeng.” The collected data from different sources were comprehensively summarized for botany, ethnopharmacology, phytochemistry, pharmacology, and toxicology of the genus *Duhaldea* DC. ChemDraw 20.0 was used to extract the chemical metabolites. The PubChem database (https://pubchem.ncbi.nlm.nih.gov) was used to confirm the chemical classifications and structures, and iPlant (https://www.iplant.cn/) was used to verify the names of the plants.

## 2 Traditional uses and ethnopharmacology

Botanical drugs have been used to treat an array of conditions by humans since ancient days (Schulz et al., 2001). *Duhaldea* DC species are widely distributed in Asia. The botanical drug uses of the genus *Duhaldea* DC are summarized in Table 1.

**TABLE 1:** Summary of the ethnobotanical uses of the genus *Duhaldea* DC (species name, plant part, country, uses, mode of administration, and associated references).

Species	Plant part used	Uses	References
*D. wissmannian*	Root	Infantile malnutritional stagnation	*Synopsis of Chinese Ethnic Medicine* (in Chinese)
*D. nervosa*	Root or whole	Treatment of rheumatic diseases such as rheumatic ostealgia, beriberi edema, and rheumatic arthritis Treatment of digestive system diseases such as dyspepsodynia, cacochylia, dyspepsia abdominal distension, chronic gastritis, and esophagus cancer Treating fractures and bone wounds Treatment of other diseases such as cold, sputum cough, ardent fever, pain in waist and lower extremities, neuralgia, and mammitis	Long (2004), *Dian Nan Ben Cao* (in Chinese)*, Yunnan Selected Chinese Materia Medica* (in Chinese)*, Yunnan Simao Selected Chinese Materia Medica* (in Chinese)*, Synopsis of Chinese Ethnic Medicine* (in Chinese)*,* and *Compendium of Chinese Traditional Medicine Resources* (in Chinese)
*D. cappa*	Leaf, root, or whole plant	Treatment of a variety of painful diseases such as rheumatic pain in waist and lower extremities, rheumatic ostealgia, toothache, dyspepsodynia, rheumatic arthralgia, and nervous headache Treatment of multiple inflammatory conditions such as kidney inflammation edema, infantile pneumonia, gastritis, vesical catarrh, amygdalitis, bronchitis, gingivitis, mammitis, and hepatitis Treatment of parasitic infectious diseases such as bilharziasis, malaria, and acariasis Treatment of other diseases injuries from falls, fractures, contusions and strains, hemorrhage, cold cough, ardent fever, itchy skin, infantile fever, neuroticism, sore and furuncle, puerperal cold, irregular menses, tuberculosis, snakebite, diarrhea, postpartum cold, and hemorrhoids	*Yunnan Selected Chinese Materia Medica* (in Chinese)*, Yunnan Simao Selected Chinese Materia Medica* (in Chinese)*, Chinese Ethnic Materia Medica Monographs* (in Chinese)*, Synopsis of Chinese Ethnic Medicine* (in Chinese)*,* and *Compendium of Chinese Traditional Medicine Resources*
*D. pterocaula*	Root	Treatment of ulcerative carbuncle pyogenic infections, tracheitis, injuries from falls, fractures, contusions and strains, deficiency of vital energy, dizziness, cough due to lung deficiency, dysentery, tinnitus, insomnia, fluster, splenic organ swelling, anemofrigid cold, dizziness, cold, and tuberculosis of bones and joints	Chunli et al. (2021), *Compendium of Chinese Traditional Medicine Resources* (in Chinese)*,* and *Synopsis of Chinese Ethnic Medicine* (in Chinese)
*D. eupatorioides*	Root	Treatment of malnutritional stagnation	*Synopsis of Chinese Ethnic Medicine* (in Chinese)

## 3 Phytochemistry

### 3.1 Preliminary phytochemical screening

Early phytochemical studies of *Duhaldea* DC. were conducted in the 1990s and revealed the presence of saponins in the sesquiterpenoids of this species (Baruah et al., 1980; Goswami et al., 1984). Large sesquiterpenoids were also recently identified in the alcohol extract of *D. wissmanniana* (Cheng et al., 2013; Cheng et al., 2014b). Other studies in subsequent years have led to the isolation and identification of phenylpropanoids, flavonoids, inositol angelates, phenolic metabolites, phenolic, monoterpenes, diterpenes, and triterpenes from acetone, chloroform, methanol, and ethanol extracts of *D. cappa* (Wang et al., 2012; Zhou, 2017). Moreover, a study on the chemical metabolites of the root of *D. cappa* resulted in the extraction and characterization of a mixture of five ceramide metabolites from this species (Guo et al., 2007). In the past few decades, liquid chromatography–mass spectrometry (LC-MS), especially ultra-high-performance liquid chromatography–high-resolution mass spectrometry (UHPLC-HRMS), has become the most powerful and reliable analytical instrument in the detection and characterization of metabolites from traditional Chinese medicine, drug, or biological samples (Wei et al., 2020). UHPLC-HRMS is an advanced form of an analytical technique used to separate and identify the complex mixture of metabolites found in botanical drugs. It is important to make generalization about the fragmentation pathways of reference metabolites using the HRMS technique to speculate the identity of potential metabolites in genus *Duhaldea* DC. (Ma et al., 2022). In the study of the chemical composition of the whole botanical drug and inflorescence of *D. nervosa*, UHPLC-Q-Exactive Orbitrap mass spectrometer and UHPLC-QTOF-MS/MS were used, and 149 chlorogenic acid derivatives and 34 metabolites were finally identified, respectively (Wei et al., 2020; Wei et al., 2022). UHPLC-Q-Exactive Orbitrap mass spectrometer and UHPLC-QTOF-MS/MS were used in the study of the chemical composition of *D. cappa*, and 68 chlorogenic acid derivatives and 12 metabolites were finally identified, respectively (Peng et al., 2017; Peng et al., 2021).

### 3.2 Chemical composition

**FIGURE 1:**
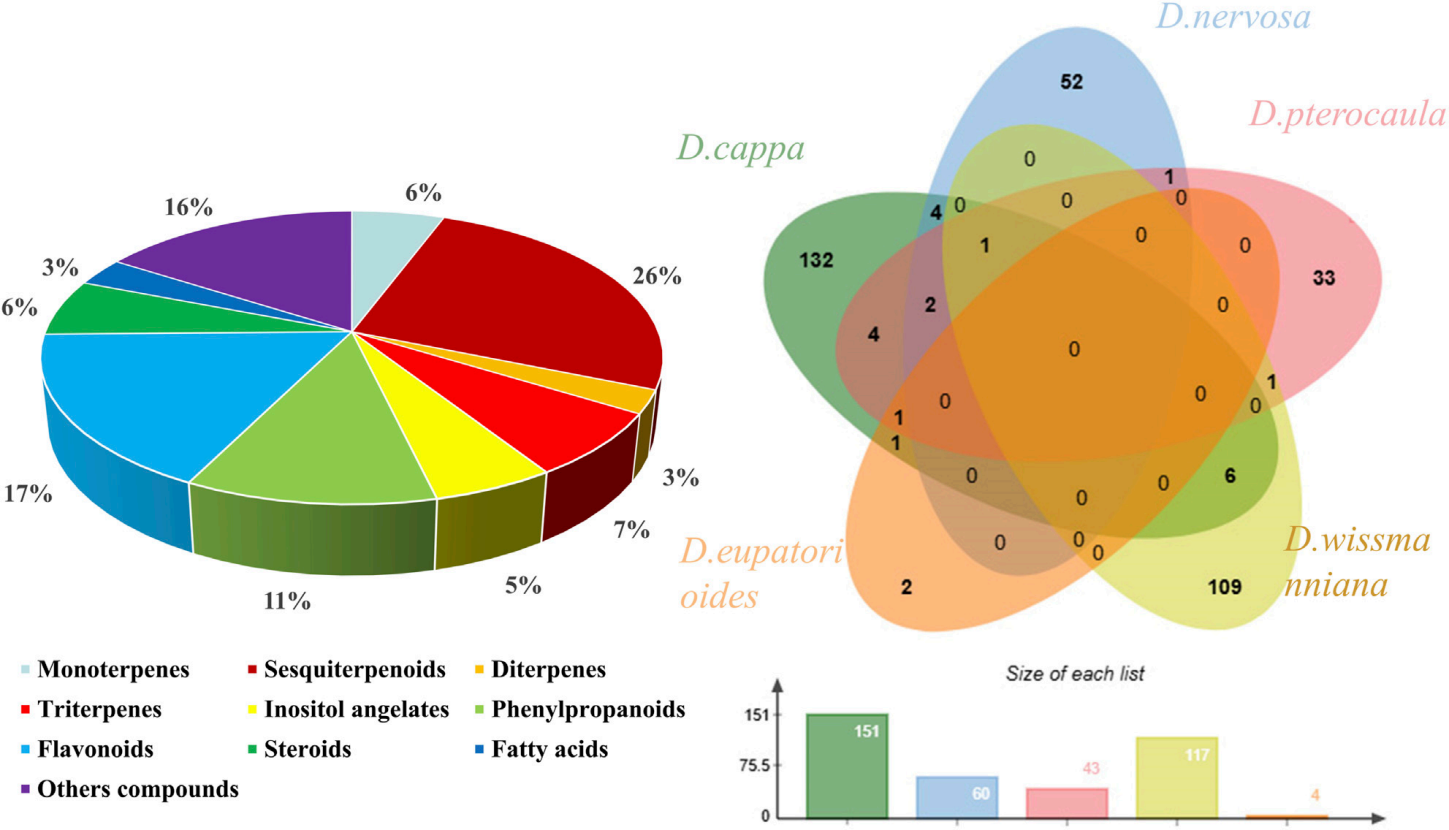
Chemical classes and proportions of phytochemical metabolites isolated and characterized from the genus *Duhaldea* DC.

Detailed phytochemical studies on *Duhaldea* DC. have revealed in an array of secondary metabolites. Many researchers, especially in the past 40 years, have discovered new metabolite structures from *Duhaldea* DC. To date, a total of 393 chemical metabolites have been reported from *Duhaldea* DC. species, including sesquiterpenoids, monoterpenes, diterpene, triterpenes, inositol angelates, phenylpropanoids, flavonoids, phenolic metabolites, and ceramide metabolites (Figure 1). The detailed information on these metabolites is summarized in Supplementary Table S1.

#### 3.2.1 Monoterpenes

**FIGURE 2:**
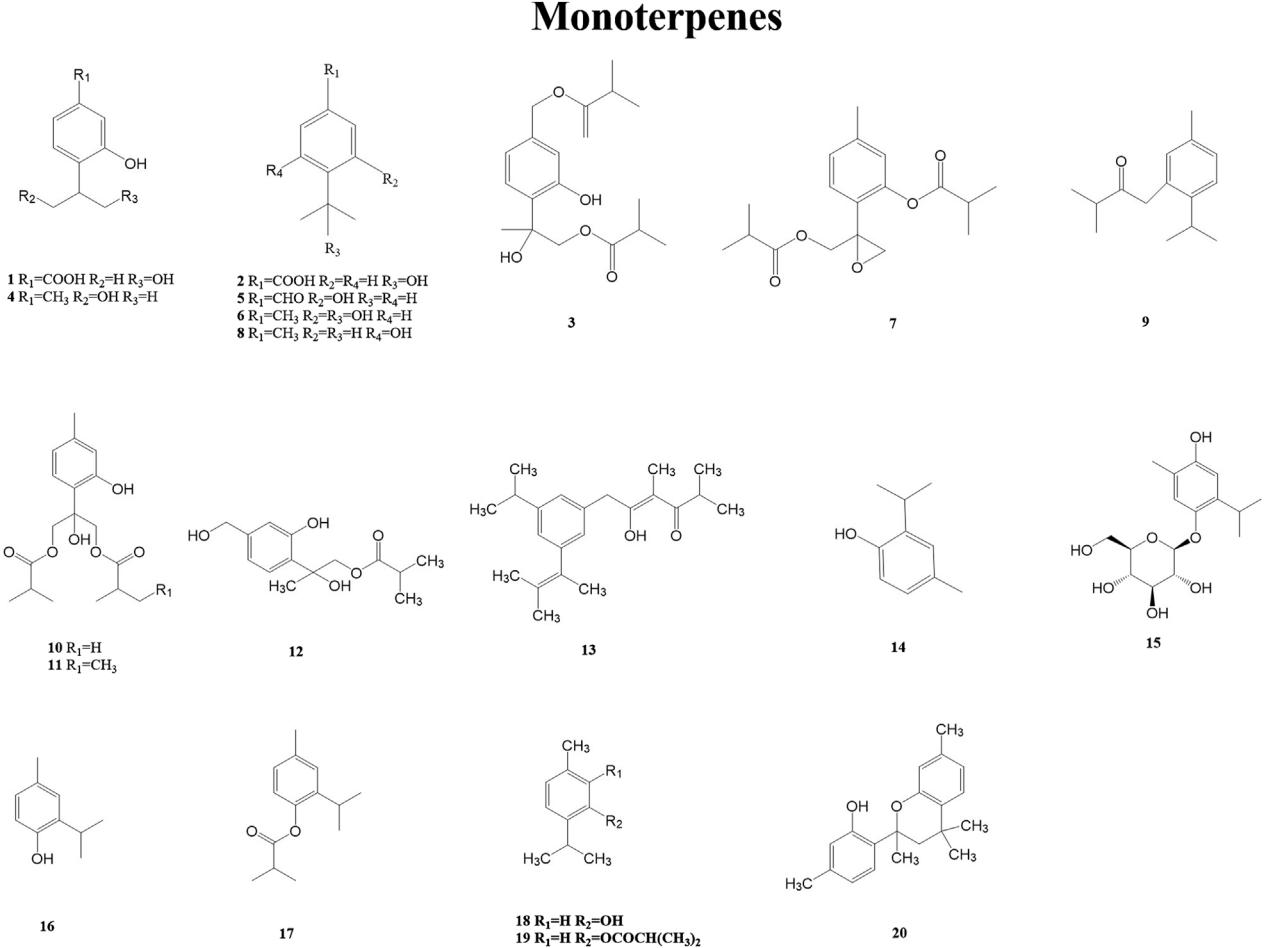
Structures of monoterpenes (metabolites 1–20).

Monoterpenes belong to a large and diverse group of naturally occurring metabolites. The basic structure of monoterpenes, or monoterpenoids, consists of two linked isoprene units. They might be cyclized and oxidized in a variety of ways. Due to their low molecular weight, many of them exist in the form of essential oils. Many monoterpenes and their derivatives have anti-inflammatory, antimicrobial, anticonvulsant, analgesic, antiviral, anticancer, antituberculosis, and antioxidant biological activities (Zielińska Błajet and Feder Kubis, 2020). A total of 20 monoterpenes (1–20) have been reported from the genus *Duhaldea* DC.*,* and Figure 2 shows their chemical structures.

#### 3.2.2 Sesquiterpenoids

**FIGURE 3:**
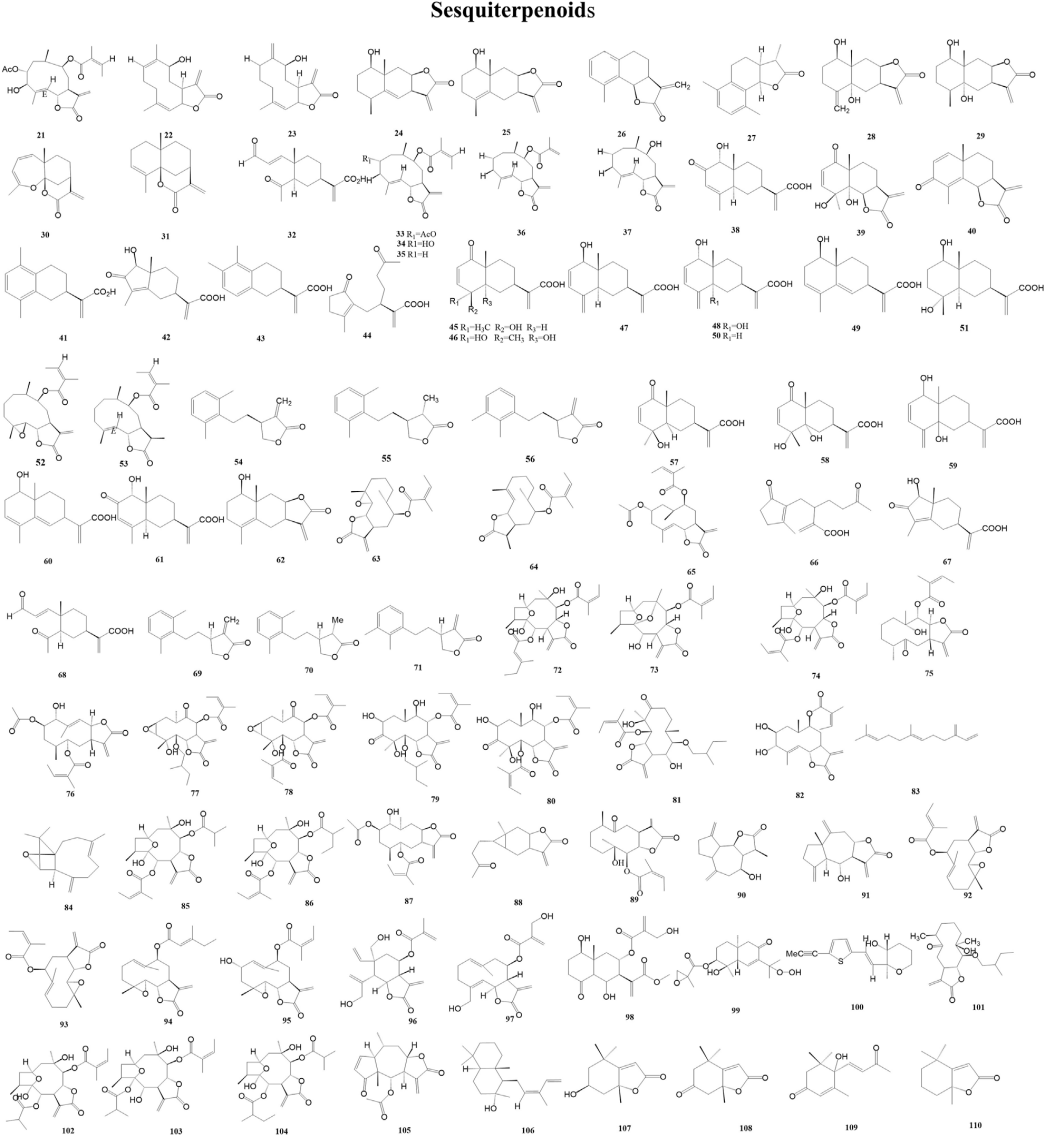
Structures of sesquiterpenoids (metabolites 21–110).

In the study of chemotaxonomy based on secondary metabolites from the Asteraceae family, sesquiterpene lactones, which are used as taxonomic markers, are a more studied class of secondary metabolites. More than 4,000 sesquiterpene lactones with around 30 different skeletons have been reported from several tribes of Asteraceae (Wu et al., 2006). A total of 90 sesquiterpenoids (21–110) have been reported from the genus *Duhaldea* DC., and Figure 3 shows their chemical structures.

#### 3.2.3 Diterpenes

**FIGURE 4:**
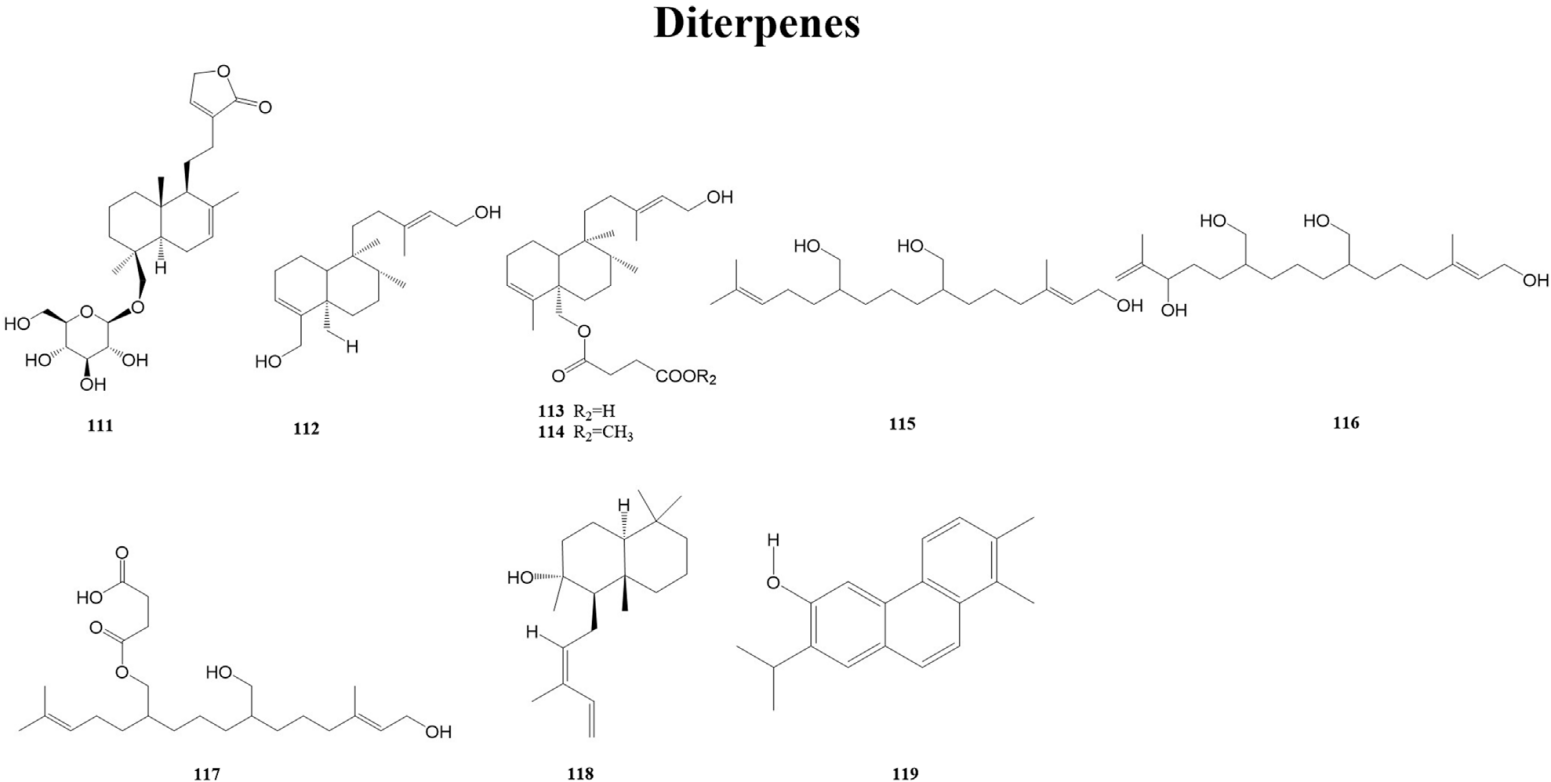
Structures of diterpenes (metabolites 111–119).

Diterpenes are the product of the mevalonic acid biosynthesis pathway. Some diterpenes have antiviral activity, such as kirkinine, *Excoecaria* toxin (anti-HIV), jiadifenoic acids JP (anti-Coxsackie virus), briaexcavatolide U, briaexcavatin L (anti-HCMV), genkwanin P, laurifolioside A (anti-HBV), linearol, isosidol (anti-HPIV-2), and debromoaplysiatoxin (anti-CHIKV) (Wardana et al., 2021). A total of nine diterpenes (111–119) have been reported from the genus *Duhaldea* DC., and their chemical structures are shown in Figure 4.

#### 3.2.4 Triterpenes

**FIGURE 5:**
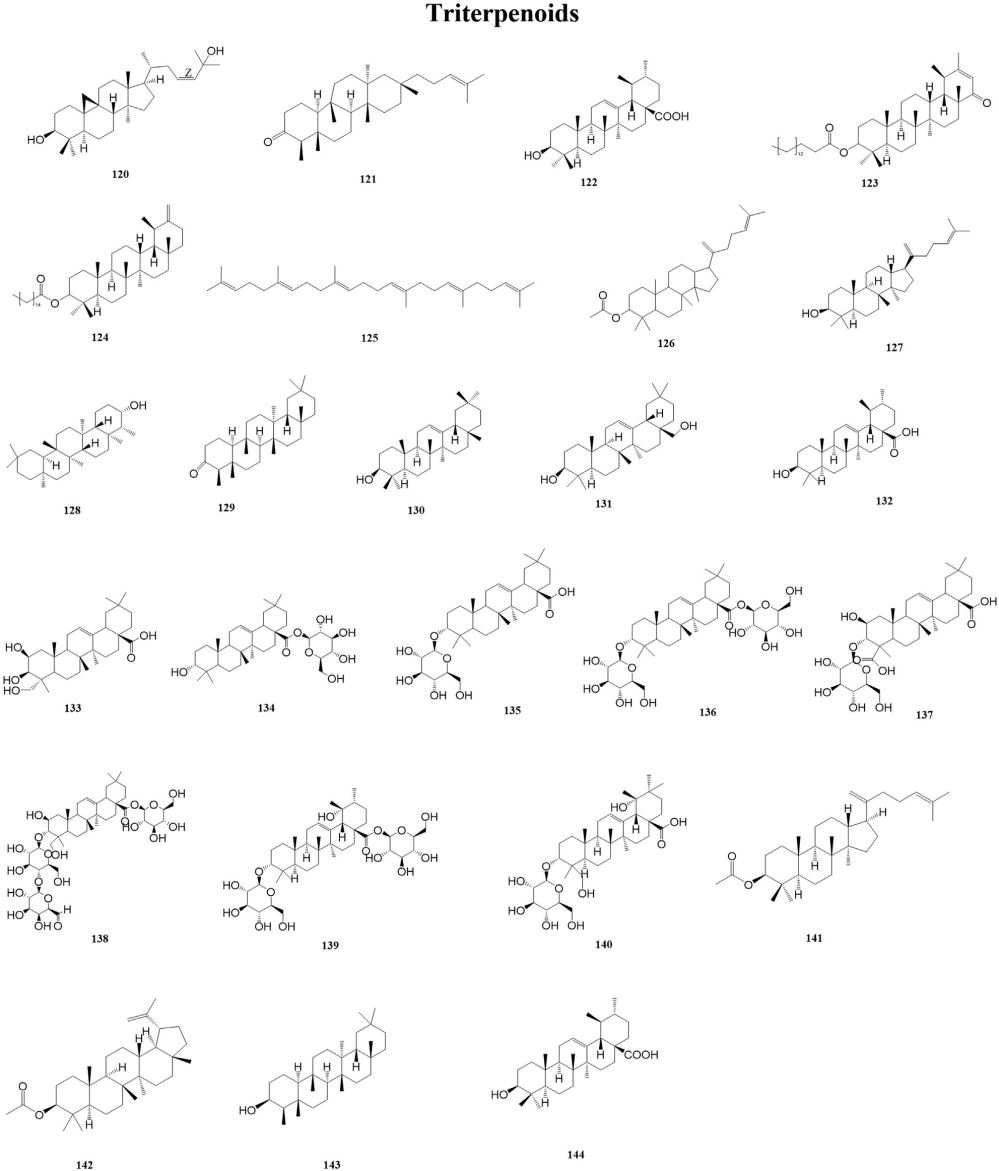
Structures of triterpenes (metabolites 120–124).

Triterpenoids are a class of terpenoids with a basic skeleton of 30 carbon atoms consisting of six isoprene units, which exist in the plant body in free form or in the form of glycosides or esters combined with sugars, and have a wide range of biochemical activities, such as anti-inflammatory and antitumorigenic activities (Gill et al., 2016; Miranda et al., 2022). At the same time, triterpenes play a vital role in the formation of structures in plant membranes, which stabilize the phospholipid bilayers in the cell membranes (Liby et al., 2007). A total of 25 triterpenoids (120–144) have been reported from the genus *Duhaldea* DC., and their chemical structures are shown in Figure 5.

#### 3.2.5 Inositol angelates

**FIGURE 6:**
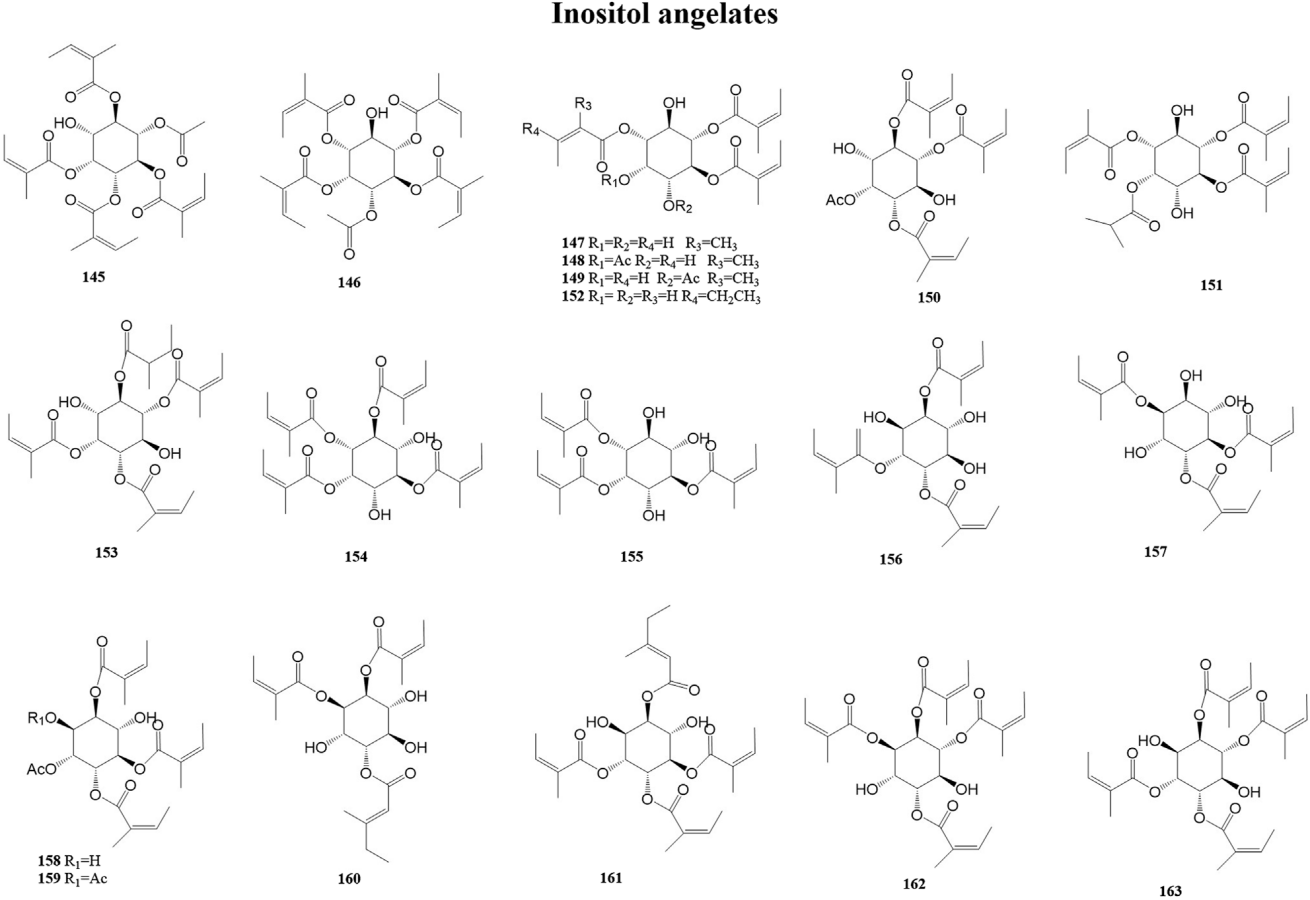
Structures of inositol angelates (metabolites 145–163).

Inositol, also known as cyclohexane hexanol, is widely distributed in animals and plants and is a growth factor for animals and microorganisms. It was first isolated from the heart muscle and liver. Muscle inositol is an essential nutrient source for birds and mammals, and a deficiency of muscle inositol can, for example, cause symptoms such as hair loss in mice and periocular abnormalities in rats. Furthermore, inositol deficiency may be involved in the pathogenesis of diseases, such as metabolic syndrome, spina bifida (a neural tube defect), polycystic ovary syndrome, and diabetes (Marine and Christophe, 2013; Kiani et al., 2021; Dorina et al., 2023). A total of 19 inositol angelates (145–163) have been reported from the genus *Duhaldea* DC., and their chemical structures are shown in Figure 6.

#### 3.2.6 Phenylpropanoids

**FIGURE 7:**
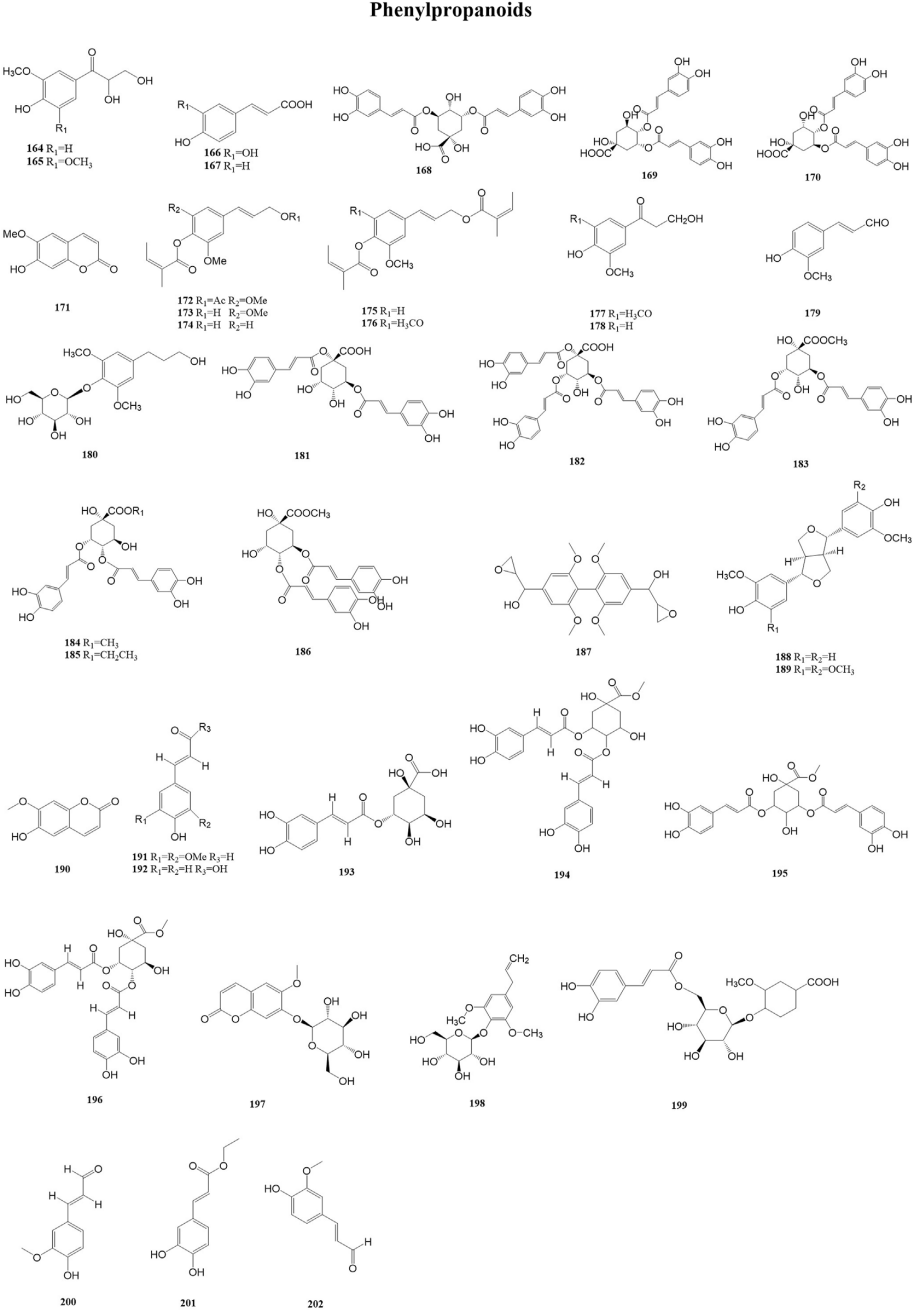
Structures of phenylpropanoids (metabolites 164–202).

Phenylpropanoids form a class of metabolites with a core structure consisting of a phenyl group attached to a three-carbon chain. This C6–C3 carbon skeleton is usually obtained from the enzymatic deamination of the aromatic amino acid phenylalanine. Phenylpropanoids, owing to their antimicrobial and antioxidant activities, are applied in pharmaceutical products as a preservative. Currently, in many countries, phenylpropanoid derivatives have been approved as food additives and active metabolites in skincare products (Neelam et al., 2020). A total of 39 phenylpropanoids (164–202) have been reported from the genus *Duhaldea* DC., and their chemical structures are shown in Figure 7.

#### 3.2.7 Flavonoids

**FIGURE 8:**
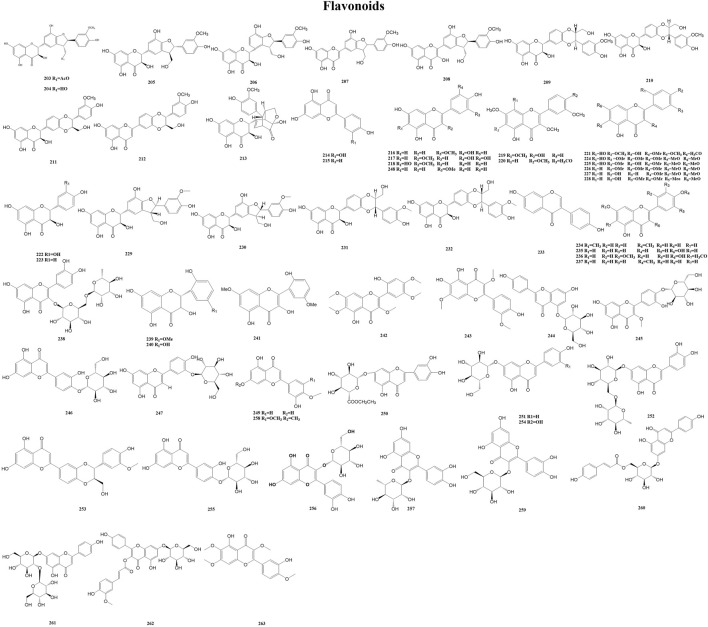
Structures of flavonoids (metabolites 203–263).

Flavonoids are a group of polyphenolic metabolites produced in plants as secondary metabolites. Flavonoids are a class of yellow pigments derived from 2-phenylchromanone as the mother nucleus, including the isomers of flavonoids and their hydrogenation and reduction products, i.e., a series of metabolites with C6-C3-C6 as the basic carbon framework. Flavonoids are widely found in fruits, vegetables, and other food crops. They have favorable biochemical effects on multiple diseases (e.g., cardiovascular disease and atherosclerosis), as well as other bioactivities (e.g., anti-inflammatory, antiviral, and antioxidant activities) (Serafini et al., 2010; Fardoun et al., 2020; Badshah et al., 2021; Shen et al., 2022). A total of 61 flavonoids (203–263) have been reported from the genus *Duhaldea* DC., and their chemical structures are shown in Figure 8.

#### 3.2.8 Steroids

**FIGURE 9:**
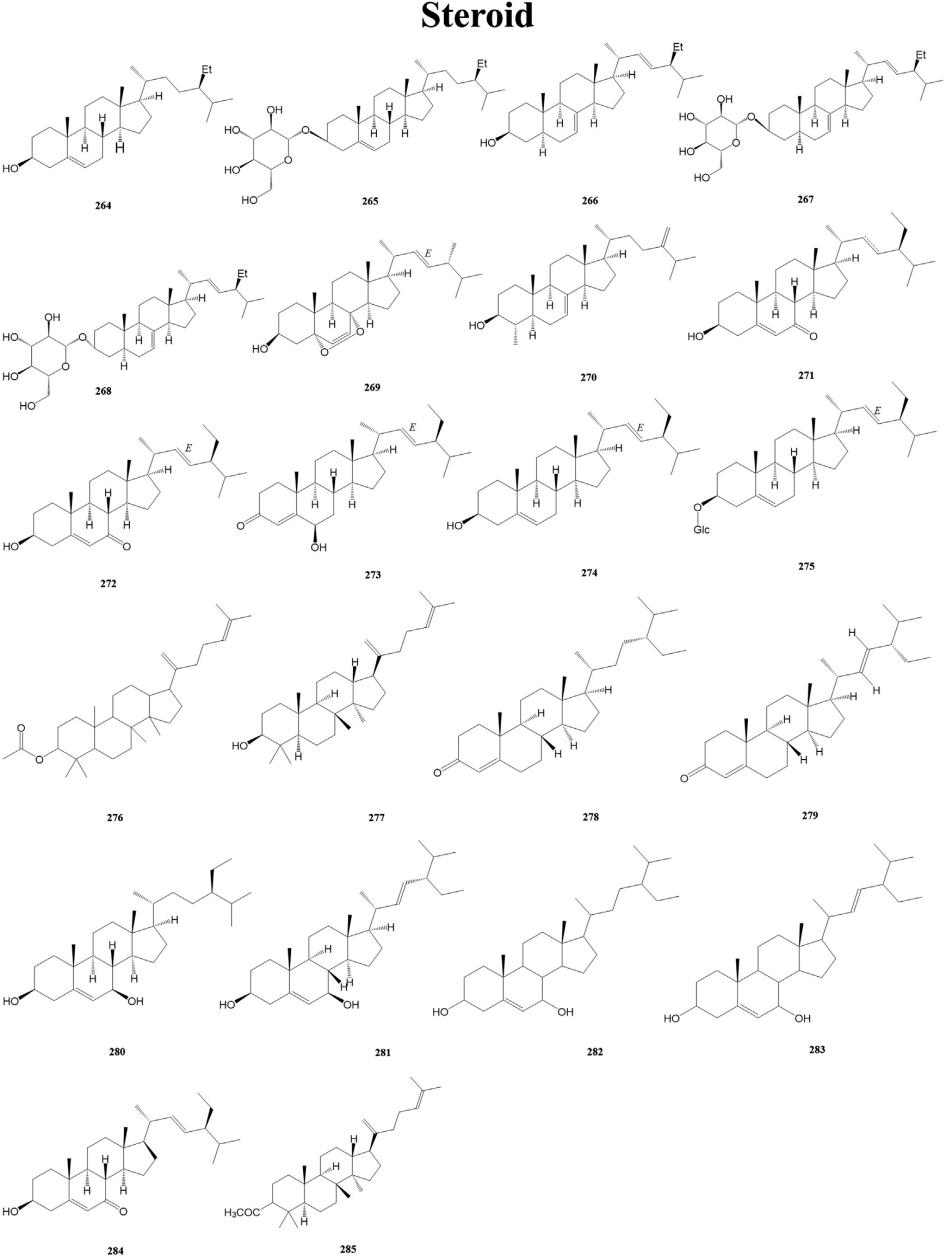
Structures of steroids (metabolites 264–285).

Steroid is a general term for a large group of cyclopentane-thickened fully hydrogenated phenanthrene derivatives that are widely distributed in living organisms. Steroids are widely known for their potent anti-inflammatory and immune-modulating activities (Neilsen et al., 2014). A total of 22 steroids (264–285) have been isolated from the genus *Duhaldea* DC., and their chemical structures are shown in Figure 9.

#### 3.2.9 Fatty acids

**FIGURE 10:**
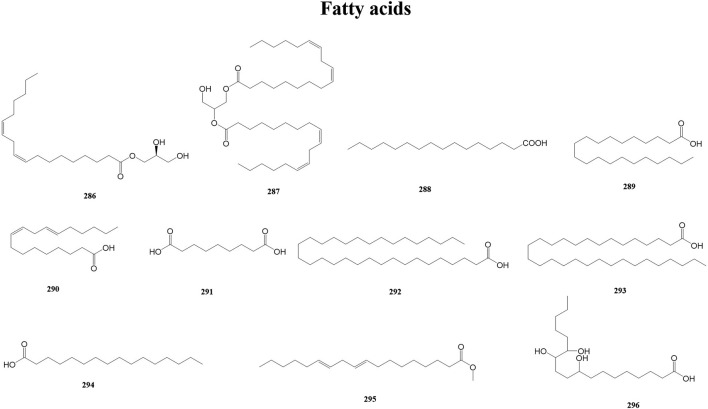
Structures of fatty acids (metabolites 286–296).

Fatty acids are carboxylic acids with a typical RCOOH structure, containing a methyl end, a hydrocarbon chain (R), and a carboxylic terminus. Fatty acids have both a systematic and a common name (e.g., octadecanoic and stearic). Fatty acids play multiple roles in humans and other organisms. At the same time, fatty acids are a substantial part of lipids, one of the three major metabolites of biological matter (along with proteins and carbohydrates) (Tvrzicka et al., 2011). A total of 11 fatty acids (286–296) have been reported from the genus *Duhaldea* DC., and their chemical structures are shown in Figure 10.

#### 3.2.10 Other metabolites

**FIGURE 11:**
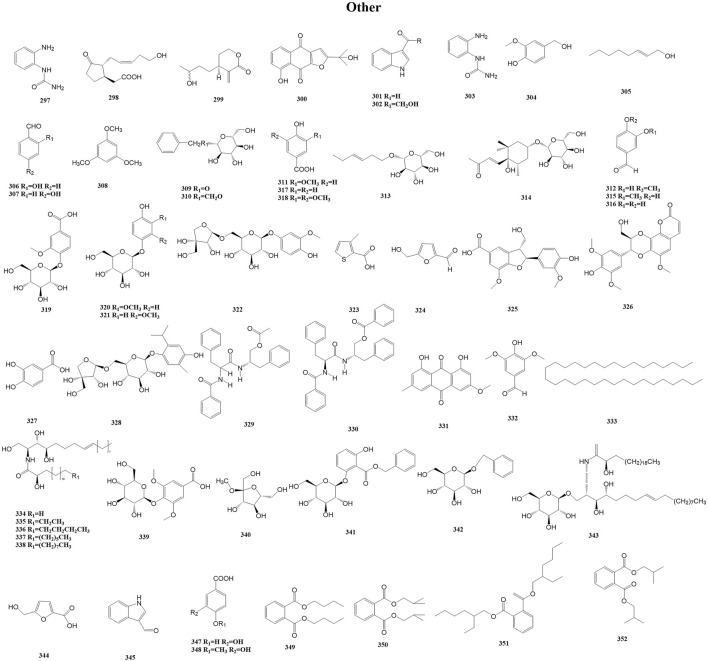
Structures of other metabolites (metabolites 297–352).

In addition to the above metabolites, *Duhaldea* DC. also contains phenolic acids, chlorogenic acid, organic acids, and polyols. These metabolites are categorized as other because they rarely exert similar pharmacological effects as a class of metabolites or they occur less frequently in the genus. For example, Wang et al. isolated and purified a novel water-soluble polysaccharide (DNP-1) from the root of *D. nervosa* via column chromatography (Wang et al., 2023). This is the only isolation of polysaccharides that we have found in our research searches of this genus. A total of 56 other metabolites (297–352) have been reported from the genus *Duhaldea* DC., and their chemical structures are shown in Figure 11.

## 4 Pharmacology

Present-day research and the study of historical texts have shown that whole plants or their respective plant parts have been used to treat or alleviate different illnesses. Currently, botanical drugs are also used in the conventional system of medicine, where plants and associated phytochemicals are being actively explored for their direct use as pharmaceutical agents. At the same time, it can be noted that the plant is used as a medicine and food, is usually used as a spice and seasoning in food, and also prevents and controls some diseases in people.

**FIGURE 12:**
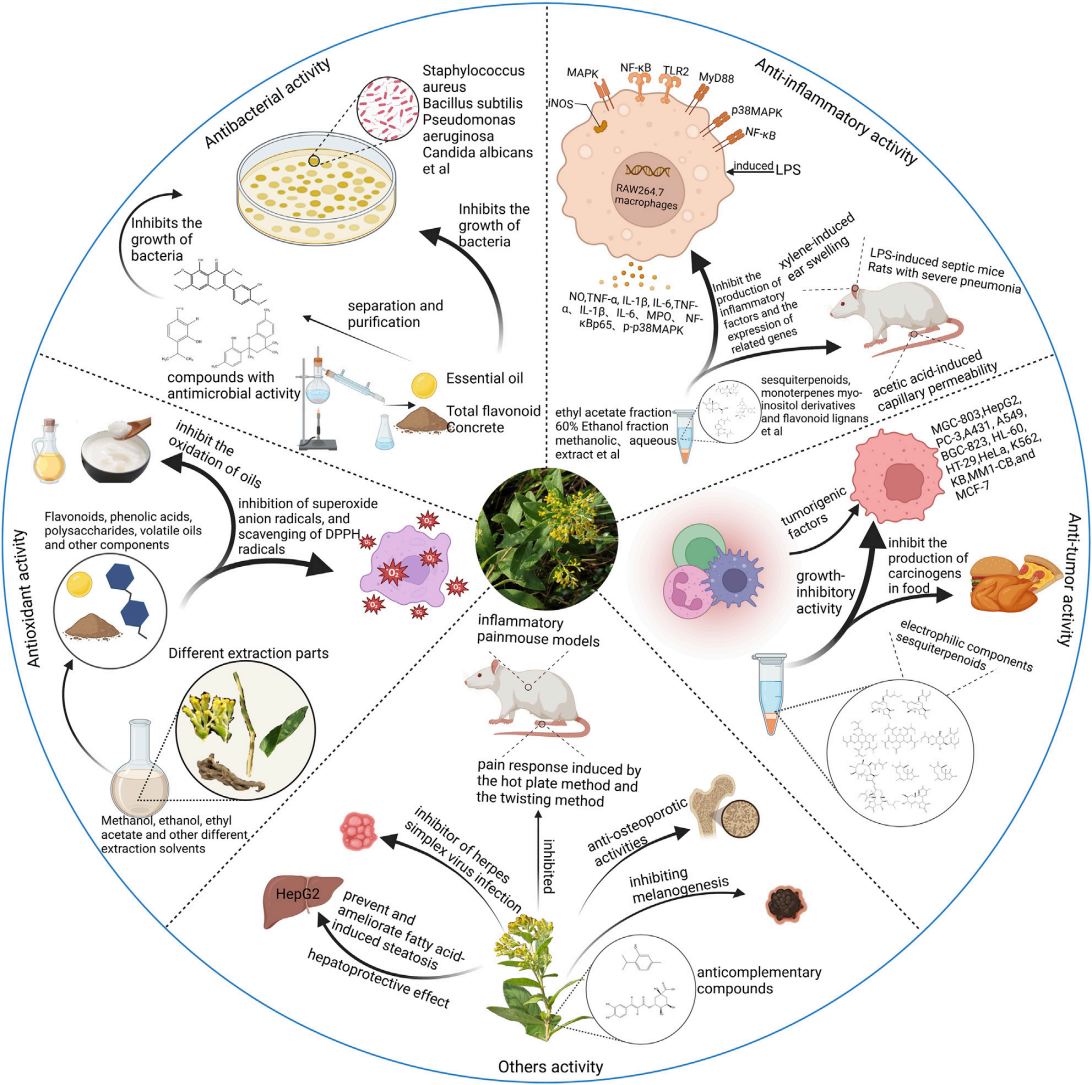
Brief description of the various pharmacological actions of the genus *Duhaldea* DC.

The genus *Duhaldea* DC. has long been used therapeutically in different countries in Asia due to its wide range of biological and pharmacological activities. The broad-spectrum ethnomedicinal uses of the different species of *Duhaldea* DC. have led to the initiation of several pharmacological investigations, such as anti-inflammatory, antioxidant, antitumor, and antibacterial activities. A brief summary of these pharmacological effects is given in Figure 12. An overview of the modern pharmacological studies on these species is detailed in the following sections.

### 4.1 Anti-inflammatory activity

Inflammation is a defensive response of the body to stimulation, manifested by redness, swelling, heat, pain, and dysfunction. In immune cells, macrophages are the main cell type involved in the inflammatory process. RAW 264.7 macrophages are a good model for screening anti-inflammatory drugs. The modern pharmacology of *Duhaldea* DC has proven to have significant anti-inflammatory effects, and its main material basis of anti-inflammation is sesquiterpenoids. A total of 21 eudesmane and germacrane derivatives were isolated from *D. wissmannian*. These isolates exhibited significant inhibitory effects on lipopolysaccharide (LPS)-induced nitric oxide (NO) production in RAW 264.7 macrophages (20, 5, 0.5, and 0.1 µM), with aminoguanidine as a positive control (Cheng et al., 2013; Cheng et al., 2014b). Wang et al. screened a total of 35 monomers isolated from *D. wissmannian*, including sesquiterpenoids, monoterpenes, and flavonoid lignans, for their inhibitory effects on NO metabolism in LPS-induced RAW 264.7 macrophages. Four concentration gradients of 50, 10, 2, and 0.4 µM were used to determine the metabolic inhibition rate and cytotoxicity of each monomer metabolite on NO, and 1*β*-hydroxy-alantolactone and inulicin were used as a positive control. The results showed that the sesquiterpenoids containing α-methylene-γ-lactone fragments exhibited strong NO metabolism inhibition, and some flavonoid lignans and myo-inositol derivatives also showed some anti-inflammatory activities (Wang, 2013). The *D. wissmannian* eudesmane-derivatized sesquiterpene, Chengwissmanolide A (IC_50_ = 0.38 μM), showed stronger or similar NO inhibitory activity to the positive drug aminoguanidine (0.20 or 0.37 μM) (Cheng, 2012). Four new sesquiterpenes, pterocaullins A−D (1–4), along with 10 known metabolites isolated from the whole plants of *D. pterocaula*, were tested for their inhibitory effects against LPS-induced NO production in RAW 264.7 macrophages using parthenolide (10 μM) as a positive control. Pterocaullin B exhibited promising anti-inflammatory activity, with an IC_50_ value of 7.03 µM (Zhu et al., 2019).

Other types of metabolites in the genus also have good anti-inflammatory activity. A new polysaccharide, DNP-1, was isolated from *D. nervosa* and inhibited the concentrations of NO, TNF-α, MCP-1, IL-2, and IL-6 pro-inflammatory factors in LPS-induced RAW 264.7 cells at concentrations of 50, 100, and 150 μg/mL (Wang et al., 2023). Wang et al. isolated 17 metabolites from *D. wissmannian,* and the anti-inflammatory activity of RAW 264.7 macrophages against LPS-induced NO production was also evaluated. Metabolites 3-acetate-1,2,4,5-tetrakis(2-methyl-2-butenoate) inositol, 2,3-dehydrosilychristin, hydnocarpin, 8-hydroxy-7,9-di-isobutyryloxythymol, and 7-hydroxy-8,9-bis(isobutyryloxy)thymol showed moderate activities, with IC_50_ values of 13.3, 19.6, 23.3, 10.8, and 10.1 μM, respectively. On the other hand, 4-acetate-1,2,3,5-tetrakis (2-methyl-2-butenoate) inositol, 23-*o*-acetylsilychristin A, isohydnocarpin, silybin B, and isosilybin A exhibited weak activities, with IC_50_ values of 32.6, 36.7, 48.2, 50.5, and 50.5 μM, respectively (Wang et al., 2013).

The metabolite (4R,5R,6S,7S,9S,10R)-9-angeloyloxy-4,5-epoxygermacra-11 (13)-en-12,6-olide, isolated from *D. wissmannian*, demonstrated potent inhibition of NO with an IC_50_ value of 1.04 ± 0.07 μM. Aminoguanidine served as the positive control and exhibited its inhibition with an IC_50_ value of 0.79 ± 0.05 μM (Cheng et al., 2014b). Maoxiucaioside A obtained from *D. nervosa*. has a significant inhibitory effect on the release of TNF-α, IL-6, and IL-1β, with IC_50_ values of 17.90 ± 2.29 μM, 19.45 ± 2.16 μM, and 18.54 ± 1.30 μM, respectively, slightly lower than those of dexamethasone (Li et al., 2023). The metabolites derived from *D. nervosa,* i.e., nervolan A, nervolan B, nervolan C, coniferyl diangelate, and sinapyl diangelate, were evaluated for their inhibitory effects on LPS-induced NO production in RAW 264.7 cells. The results demonstrated that these metabolites exhibited mild inhibitory activities against NO production, with IC_50_ values of 33.31, 15.43, 21.32, 16.19, and 33.56 μM, respectively (Yan et al., 2010). The five known analogs, isolated from *D. cappa*, exhibited anti-inflammatory activities against the production of NO in RAW 264.7 cells stimulated by LPS. Their IC_50_ values ranged from 7 to 23 μM, with celecoxib (IC_50_ 1.6 μM) serving as the positive control (Wu et al., 2015).

The *D. cappa* methanolic extract (75 and 150 mg/kg) showed reduction in the rat paw edema, significant inhibition of the cotton pellet-induced granulomas in rats, and potential immunomodulatory activity in all the assays performed (Jyoti et al., 2017). The *D. cappa* root ethanol extract (5 g/kg) significantly inhibited xylene-induced ear swelling and acetic acid-induced capillary permeability in mice and has certain anti-inflammatory effects (Mo et al., 2012), and 60% ethanol fraction of *D. cappa* is the main anti-inflammatory constituent (Gong et al., 2018; Wang et al., 2018). The *D. cappa* aqueous extract (24 g/kg) also improved the inflammatory response of severe pneumonia rats, which may be related to the inhibition of the TLR2/MyD88/NF-*κ*B signaling pathway (Aizaizi et al., 2018). The *D. cappa* aqueous extract (24 g/kg) can improve inflammation of severe pneumonia induced by *Klebsiella pneumoniae* by blocking p38 MAPK and NF-*κ*Bp65 signaling pathways (Li, 2017).

### 4.2 Antitumor activity

Tumors are formed by the proliferation of local tissue cells under the action of various tumorigenic factors. According to the cell characteristics of tumors and the degree of harm to the body, tumors are divided into benign tumors and malignant tumors. Cancer, a malignant tumor, has become the second leading fatal disease after cardiovascular and cerebrovascular diseases and seriously threatens human health and life. Modern pharmacological studies have shown that *Duhaldea* DC. has certain anticancer effects, and its main anticancer substance is sesquiterpenoids. Cheng et al. evaluated all 21 isolates; these isolates showed no significant toxicity in RAW 264.7 macrophages at concentrations up to 20 μM. Metabolites (5*R*,7*R*,10*S*)-4,5-epoxy-4,5-secoeudesma-1,3-dien-12,5-olide, 3-oxoeudesma-1,4,11 (13)-trien-12,6β-olide, haageanolide, and 11,13-dehydroisohyposantonin had strong inhibitory effects on NO, with IC_50_ values of 0.68, 0.68, 0.97, and 0.65 μM, respectively. Moreover, the cytotoxicity assay in four human tumor cell lines suggested that new metabolites (5*R*,7*R*,10*S*)-4,5-epoxy-4,5-secoeudesma-1,3-dien-12,5-olide, (2*R*,4*E*,6*R*,7*S*,9*S*,10*R*)-2-acetoxy-9-angeloyloxygermacra-4,11(13)-dien-12,6-olide, 4E-9β-angeloyloxy-2α-hydroxy-7α,10αH-germacra-4,11(13)-dien-12,6α-olide (4*E*,6*R*,7*S*,9*S*,10*R*)-9-angeloyloxygermacra-4,11 (13)-dien-12,6-olide, 4*E*-9β-methacryloxy-7α,10αH-germacra-4,11 (13)-dien-12,6α-olide, 4*E*-9β-hydroxy-7α,10αH-germacra-4,11 (13)-dien-12,6α-olide, and 3-oxoeudesma-1,4,11 (13)-trien-12,6β-olide showed strong toxicity against HepG2, PC-3, and MGC-803 cells (Cheng et al., 2013). Five germacrane-type sesquiterpene lactones (ineupatolide D, ineupatolide E, dvaricin B, nepalolide C, and inculacappolide) were isolated from *D. cappa* showed moderate inhibitory effects on A431, A549, BGC-823, HL-60, HT-29, and MCF-7 cancer cell lines with IC_50_ values ranging from 2.1 to 36.3 µM, and doxorubicin was used as the positive control (Wu et al., 2017)*.* The cytotoxicity assay showed that inulacappolide has anti-proliferative effects against human cervical cancer (HeLa), human leukemia (K562), and human nasopharyngeal carcinoma (KB) cell lines, with IC_50_ values of 1.2 mM, 3.8 mM, and 5.3 µM, respectively (Xie et al., 2007). The sesquiterpene metabolite ineupatorolide B, isolated from *D. cappa*, exhibited potent growth-inhibitory activity against HeLa cells, whereas its activity against MM1-CB melanoma cells was weaker. The mechanism by which ineupatorolide B exerts its growth-inhibitory effects may involve the activation of PKCα, leading to an enhancement of the retrotranscriptional activation capacity of NFAT (Mei et al., 2015).

As a medicinal plant, this genus not only serves as a flavorful seasoning but also inhibits the production of carcinogens in food. Cheng et al. discovered that the electrophilic metabolites act as creatinine inhibitors to reduce the generation of heterocyclic aromatic amines. Their research reveals that the rhizome of *D. nervosa*, when used as a spice, can inhibit the production of carcinogens in food (Cheng et al., 2023). *D. nervosa* shows greater potential as a functional food for cancer prevention and anticancer effects. Under the condition of simulated gastric fluid *in vitro*, the stem and leaf extracts of *D. nervosa* can effectively remove nitrite and block the production of nitrosamines (Yuan et al., 2021).

### 4.3 Antioxidant activity

An increasing body of research indicates that antioxidants play a crucial role in mitigating the effects of aging as free radicals and oxidants contribute to cellular and tissue degradation, impair metabolic functions, and are associated with various health issues. Cheng et al. compared the chemical space and antioxidant activities of ethanol extracts from different parts of *D. nervosa*. Findings support the traditional use of its roots and indicate thymol di-isobutyrate as a major functional factor. The results showed that significant correlations and extracts upregulated the mRNA expression of antioxidant response actors in H_2_O_2_-challenged HepG2 cells, hence also cueing the potential antioxidant activity of other parts (Cheng et al., 2020). He et al. compared the antioxidant effects of ultrasonic alcoholic extracts from different parts of the plant through *in vivo* and *ex vivo* experiments. *In vitro*, the extract amount was positively correlated with the clearing effect of free radicals at certain drug concentrations, with VC as the positive drug. *In vivo*, control and aging model groups had different treatments, while the experimental group had low, medium, and high (300, 600, and 1,200 μg g^−1^·d^−1^)-dose intragastric administrations. The results showed good free radical scavenging and antioxidant capacity, with the underground part having a significantly higher antioxidant effect than the aboveground part (He et al., 2016). Yang et al. investigated the antioxidant activity of polysaccharides from above-ground and below-ground parts of *D. nervosa* and total flavonoid of *D. nervosa* and found that the reducing ability of polysaccharide and flavonoid, the inhibiting ability of superoxide anion free radical, and the scavenging ability of the DPPH radical were greater than that of para-rutin at high concentration, and the antioxidant ability was higher than that of rutin with the increase in concentration. They show a high scavenging rate of hydroxyl radicals (Tao et al., 2015; Tao et al., 2016). Kalola et al. used the DPPH radical scavenging assay, superoxide radical scavenging assay, measurement of reducing power, and measurement of the effect on lipid peroxidation in rat liver homogenates, and butylated hydroxytoluene, gallic acid, and ascorbic acid were used as the positive control. The results showed that the *D. cappa* methanolic extract and the ethyl acetate-soluble fraction exhibited higher antioxidant activity (Kalola and Shah, 2006). The volatile oil (310–520 mg/L) of *D. cappa* has some scavenging ability for both hydroxyl radicals (positive control: mannitol and thiourea) and superoxide anion radicals (positive control: VC), but the scavenging activity of hydroxyl radicals is stronger (Liu et al., 2009a). *D. nervosa* extracts (0.05 g/g) can also effectively inhibit the oxidation of oils (Liu et al., 2011).

### 4.4 Antibacterial activity

Antimicrobial activity is an important indicator for screening natural metabolites for their potential to become antibiotics. Ethanol extracts (10 μg/mL) and petroleum ether extracts (10 μg/mL) of *D. nervosa* significantly inhibited *Staphylococcus aureus* and *Bacillus subtilis* (Liu et al., 2011). The essential oil derived from *D. cappa* comprises approximately 50% sesquiterpene hydrocarbons. Streptomycin (30 μg/disk) and erythromycin (15 μg/disk) were used as positive controls, while hexane was taken as a negative control, and at the concentration of 1,000 μL/mL, it has a significant inhibitory effect on *Enterococcus faecalis*, *Klebsiella pneumoniae*, *Xanthomonas viripennis*, and *B. subtilis* (Priydarshi et al., 2016). The antimicrobial activity of the flavonoid extracts from *D. cappa* was studied against nine microorganisms. The results showed that the minimal inhibitory concentration (MIC) of the *D. cappa* flavonoids to *Sarcina lutea* was the best with a ratio of 0.0039 g/mL; the MIC of *E. faecalis*, *B. subtilis,* and *S. aureus* was 0.1250 g/mL; the MIC of *Escherichia coli*, *Salmonella typhimurium*, and *Salmonella* paratyphi A was 0.5000 g/mL but no obvious inhibitory effect on *Proteus vulgaris* and *Candida albicans* (Liu W. et al., 2010). Liu et al. showed that the MIC values of roots, stems, and leaves against *S. aureus* were 15.63, 62.5, and 62.5 mg/mL, respectively, while the MIC values of roots, stems, and leaves against *Pseudomonas aeruginosa* were 15.63, 31.25, and 62.5 mg/mL, respectively (Liu et al., 2009b). The antimicrobial effects of different extracts from different parts of *D. cappa* on different bacteria were compared, among which the glacial acetic acid extract had the best antimicrobial effect, the root and leaf extracts had better antimicrobial effects than the stem extract, and the extracts had better inhibitory effects on Gram-positive than Gram-negative bacteria (Liu S. et al., 2010). The growth of 15 plant pathogenic fungi and 4 bacteria could be inhibited by 100 mg/mL of thymol isolated from *D. cappa* (Xie et al., 2012). By comparing the various extraction and separation fractions of the ethanol extract of *D. cappa*, the chloroform:acetone 10:0 fraction of the ethyl acetate extract exhibited the most significant antibacterial activity at a concentration of 2.0 mg/mL, followed by the ethyl acetate extract and the chloroform:acetone 9:1 fraction (Li et al., 2020).

### 4.5 Others’ activity

The electrophilic metabolites (0.5, 2.0, and 8.0 μg/mL) of *D. nervosa* attenuated hepatic steatosis in FFA-treated HepG2 cells. Studies suggest that it improved hepatic lipid metabolism disorder, Krebs cycle activity, oxidative phosphorylation, and the cellular and mitochondrial redox status. Additionally, 10-isobutyryloxy-8,9-epoxythymol isobutyrate activates the Nrf2-ARE signaling pathway by upregulating Nrf2 expression and promoting Nrf2 nuclear translocation (Cheng et al., 2022). Dong et al. found that the ethyl acetate fraction of *D. nervosa* (0.5–2.0 μg/mL) effectively reduced hepatic lipid accumulation and ROS production, lowered TG and TC levels, and enhanced antioxidant enzyme activity. The depletion of electrophilic components reduced its efficacy and regulatory effects on lipid metabolism and redox-related gene expression (Dong et al., 2020).

The ethyl acetate fraction of *D. pterocaula* (40 mg/kg, 100 μL) demonstrated potent analgesic effects in inflammatory pain mouse models and caused no anti-nociceptive tolerance (Huang et al., 2021b). The alcoholic extract of the *D. cappa* root (5 g/kg) significantly inhibited the pain response induced by the hot plate method and the twisting method in mice and had some analgesic effects (Mo et al., 2012). Kiran et al. and Yupei et al. developed a hepatotoxicity model through the intraperitoneal injection of carbon tetrachloride suspended in sterile oil or D-GalN. Silymarin at a dosage of 100 mg/kg and bifendate at 0.2 g/kg were identified as positive control drugs. The dosages for the water extract of *D. cappa* were administered at 9.8 g/kg, 19.5 g/kg, and 39.0 g/kg and 200 mg/kg and 400 mg/kg, and the effects on hepatotoxicity were assessed after a 10-day treatment period. The aqueous extract of *D. cappa* chrysanthemum showed a hepatoprotective effect against carbon CCl4 and D-GalN-induced hepatotoxicity in rats (Kiran et al., 2017; He et al., 2019). Yang et al. screened a PXR-HepG2 cell model with high expression of the pregnane X receptor. A negative control group (DMSO group, 0.1%), a drug treatment group (25, 50, and 100 mg/L), and a positive control group were treated with the pregnane X receptor agonist rifampicin (10 μmol/L) for 24, 48, and 72 h, respectively. Yang et al. showed that *D. cappa* can affect the expression of P450 enzymes in primary rat hepatocytes and HepG2 cells with high expression of the pregnane X receptor (Chang et al., 2020).

The methanol and dichloromethane extracts of *D. cappa* are potent inhibitors of herpes simplex virus infection *in vitro*. The *D. cappa* methanol extract exhibited significantly higher anti-herpes simplex virus (HSV) activity than the *D. cappa* dichloromethane extract as it inhibited more than 50% of the virus. The 50% effective doses of the *D. cappa* methanol extract against HSV-1 and HSV-2 were determined to be 720.1 ± 32.7 μg/mL and 529.2 ± 5.2 μg/mL, respectively (Nikomtat et al., 2011). The 12 isolated triterpenoids were screened for their anti-osteoporotic activities, and the metabolites oleanoic acid 3-O-(β-D-glucopyranosyl)-28-O-β-D-glucopyranosyl ester and 2β-hydroxyolean-3-O-(β-D-glucopyranosyloxy)-12-en-23, 28-dioic acid showed good anti-osteoporotic activities with IC_50_ values of 86.3 and 51.6 μg/mL, respectively, and their inhibitory effects were slightly lower than those of the positive control, teriparatide (45.6 μg/mL) (Tai et al., 2014). Fujita et al. studied the mechanism of action of 5–15 µM inulavosin isolated from *D. nervosa* (Compositae) in inhibiting melanogenesis, reducing the melanin content without affecting the enzymatic activities or the transcription of tyrosinase or Tyrp1 in B16 melanoma cells (Hideaki et al., 2009). Wen et al. evaluated the anti-complementary effects of blossoms of *D. nervosa* extracts based on the classical pathway. Their results showed that chlorogenic acid, 3,5-dicaffeoylquinic acid, 1,5-dicaffeoylquinic acid, and thymol were the major anti-complementary metabolites in the blossoms of *D. nervosa* (Wen et al., 2019).

## 5 Conclusion and future perspectives

The genus *Duhaldea* DC. has been used in traditional medicine to treat fractures, bone wounds, carbuncle and poison, bronchitis, bruises, giddy with hypertension of qi, and lung deficiency cough. Five species of the genus *Duhaldea* DC. have been reported to have various applications in traditional systems of medicine in several Asian countries. However, ethnobotanical data are sometimes hard to find, and it is difficult to access sources. Therefore, although the authors have made great efforts to cover the available literature as rigorously as possible, there may be possibilities that some publications, reports, or books on traditional medicinal uses of the genus *Duhaldea* DC. escaped our exploration. Available data indicate that over 352 metabolites have been identified from this genus, including terpenoids, flavonoids, phenylpropanoids, inositol angelates, chalcones, phenolics, and ceramide metabolites. The genus *Duhaldea* DC. is associated with various pharmacological activities, including anti-inflammatory, antimicrobial, anti-osteoporotic, anticancer, and antioxidant activities. Despite the fruitful phytochemical and pharmacological studies on genus *Duhaldea* DC., there are still several key issues to be resolved regarding the need for further development of genus *Duhaldea* DC.

At present, the genus *Duhaldea* DC. comprises 15 species widespread in Central, East, and Southeast Asia. However, only three (*D. wissmannian*, *D. nervosa*, and *D. cappa*) of the plants in the genus have been more extensively studied. In the future, more attention needs to be paid to underexplored plants in the study of this genus; these underexplored plant species could be promising candidates for further research.

Regrettably, aside from the systematic characterization and separation of plant metabolites conducted by Wang and Cheng et al. on *D. wissmannian* (Cheng, 2012; Wang, 2013), most other phytochemical investigations related to this species remain relatively fragmented. Phytochemistry seeks to identify valuable new plant metabolites. There exist considerable gaps in the study of the chemistry within this genus, making the systematic characterization and separation of its metabolites an urgent priority. Simultaneously, plant metabolites encompass not only various small-molecule metabolites but also macromolecular metabolites such as polysaccharides and peptides, which exhibit remarkable pharmacological activities. However, it is disheartening to note that among the extensive studies conducted on this genus, only Wang et al. realized this aspect (Wang et al., 2023).

Although advancements have been achieved in understanding the chemical and pharmacological characteristics of the genus *Duhaldea* DC., several concerns remain. Notably, the majority of pharmacological investigations on plant metabolites have been conducted exclusively *in vitro*, leading to a low confidence level regarding the validity of such simplistic *in vitro* studies demonstrating activity. Likewise, certain pharmacological assessments of extracts from this genus have only confirmed their efficacy *in vitro*. The current study mainly focused on the pharmacological activity profile of the extracts and isolated metabolites of the genus *Duhaldea* DC, while the mechanism of action was less studied, and targets and channels of action of the active metabolites corresponding to diseases were not scientifically elucidated. Modern scholars can consider using network pharmacology, data mining, and virtual screening to predict the targets, receptors, and pathways of their chemical metabolites related to pharmacological activities. At the same time, the molecular mechanisms and relationships between the active metabolites of the genus and the potential pharmacological activities are validated through high-quality and well-designed *in vivo* and *in vitro* and clinical studies.

In terms of pharmacological effects, most of them have focused on the anti-inflammatory, antitumor, antioxidant, and other effects, but at present, there are few modern pharmacological studies of the traditional effects of FI in the treatment of diseases. These ignore the modern pharmacological interpretation of traditional applications. In the future, pharmacologic studies of the genus *Duhaldea* DC can focus on indications in its traditional applications and should also provide a modern pharmacological explanation for the traditional application of the genus *Duhaldea* DC. At the same time, it should be noted that the plants of the genus *Duhaldea* DC are used as both medicine and food, and the pharmacological effects of the application in food and the effect on the inhibition of some disease-causing factors in food, as well as the prevention and control of some diseases.

Finally, *Duhaldea* DC species possess various biological activities, which can be applied to clinical medicine with further research. In addition, with the advancements observed recently in analytical techniques and quality control methods, among which the improvement and update in chromatography techniques and molecular identification methods, it is inevitable that new quality markers and quality control measures may be adopted for the better quality assessment of traditional botanical drug medicine in the future.

Plants of the genus *Duhaldea* DC as traditional plant medicines have made outstanding contributions in treating diseases and maintaining physical health. In this study, the ethnopharmacology, phytochemistry, and pharmacological effects of the genus *Duhaldea* DC were systematically elaborated to obtain a comprehensive understanding of the genus *Duhaldea* DC and reveal the shortcomings of the current research studies. In response to these shortcomings, this study provides guidance for future research on the genus *Duhaldea* DC, providing an effective scientific basis for expanding the pharmacological effect of *Duhaldea* DC, explaining the traditional application of *Duhaldea* DC, developing new drugs rationally, ensuring drug safety, controlling drug quality, and adapting *Duhaldea* DC to clinical application.
