# Supplementary material for: A review on ethnobotany, phytochemistry, and pharmacology of the genus Duhaldea DC
Source: Front Pharmacol. 2024 Nov 15;15:1479963. doi: 10.3389/fphar.2024.1479963 (PMC11604426; doi:10.3389/fphar.2024.1479963)
Supplement: Supplementary file 2 [file Table1.DOCX]

Supplementary Material

# Supplementary Tables

Table S1. Chemical compounds isolated from the genus Duhaldea.

| S/  No | Chemical Class | Chemical Compounds | Species | Plant part | Reference |
| --- | --- | --- | --- | --- | --- |
| 1 | monoterpenes | 3-hydroxy-4-(2-hydroxy-1-methylethyl) benzoic acid | *D. wissmanniana* | Aerial part | (wang, 2013; Wang et al., 2013) |
| 2 | monoterpenes | 4-(1-hydroxy-1-methylethyl) benzoic acid | *D. wissmanniana* | Aerial part | (wang, 2013; Wang et al., 2013) |
| 3 | monoterpenes | 8-hydroxy-7,9-di-isobutyryloxythymol | *D. wissmanniana* | Aerial part | (wang, 2013; Wang et al., 2013) |
| 4 | monoterpenes | 9-hydroxythymol | *D. wissmanniana* | Whole plant | (Cheng, 2012) |
| 5 | monoterpenes | 3-hydroxy-4-isopropylbenzaldehyde | *D. wissmanniana* | Whole plant | (Cheng, 2012) |
| 6 | monoterpenes | 8-hydroxythymol | *D. wissmanniana* | Whole plant | (Cheng, 2012) |
| 7 | monoterpenes | 8,9-epoxy-10-isobutyryloxy-thymol-isobutyrate | *D. wissmanniana* | Whole plant | (Cheng, 2012) |
| 8 | monoterpenes | thymol | *D. cappa*  *D. nervosa* | Aerial part  Root  Stem | (Bohlmann et al., 1982; Duoging et al., 1997; Xie Huiting, 2012) |
| 9 | monoterpenes | thymyl isobutyrate | *D. cappa*  *D. nervosa* | Aerial part  Root | (Bohlmann et al., 1982; Duoging et al., 1997) |
| 10 | monoterpenes | 8-hydroxy-9,10-diisobutyryloxythymol  7-Hydroxy-8,9-bis(isobutyryloxy)thymol | *D. pterocaula*  *D. wissmanniana* | Whole plants  Aerial part | (Zhu et al., 2019)  (Wang, Zhang et |
| 11 | monoterpenes | 8-hydroxy-9-isobutyryloxy-10-(2-methylbutyryl) thymol | *D. pterocaula* | Whole plants | (Zhu et al., 2019) |
| 12 | monoterpenes | 7,8-dihydroxy-isobutyryl thymol | *D. nervosa* | Root | (Duoging et al., 1997) |
| 13 | monoterpenes | 2, 4-dimethyl-6 (3' -methyl-isobutyl-5 '-isopropyl) -phenyl-3, 5-hexadione | *D. nervosa* | Root | (Duoging et al., 1997) |
| 14 | monoterpenes | isothymol | *D. cappa* | Root | (Wu et al., 2010) |
| 15 | monoterpenes | zataroside A | *D. cappa* | Root | (Wu, Shan et al. 2010) |
| 16 | monoterpenes | 4-methyl-2-isopropyl phenol | *D. cappa* | Aerial parts | (Bohlmann et al., 1982) |
| 17 | monoterpenes | 4-methyl-2-isopropyl phenylacetate | *D. cappa* | Aerial parts | (Bohlmann et al., 1982) |
| 18 | monoterpenes | thymol | *D. nervosa* | Root | (Yoshida, Mori et al. 1995) |
| 19 | monoterpenes | thymol isobutylate | *D. nervosa* | Root | (Yoshida, Mori et al. 1995) |
| 20 | monoterpenes | inulavosin | *D. nervosa* | Root | (Yoshida, Mori et al. 1995) |
| 21 | Sesquiterpenes | inulacappolide | *D. wissmanniana*  *D. cappa* | Whole plant  Aerial parts | (Xie et al., 2007a; Wang et al., 2012; Cheng et al., 2013) |
| 22 | Sesquiterpenes | haageanolide | *D. wissmanniana* | Whole plant | (Cheng, Zhang et al. 2013) |
| 23 | Sesquiterpenes | maroniolide | *D. wissmanniana* | Whole plant | (Cheng, Zhang et al. 2013) |
| 24 | Sesquiterpenes | 1β-hydroxyalantolactone | *D. wissmanniana* | Whole plant | (Cheng, Zhang et al. 2013) |
| 25 | Sesquiterpenes | ivangustin | *D. wissmanniana* | Whole plant | (Cheng, Zhang et al. 2013) |
| 26 | Sesquiterpenes | 11,13-dehydroisohyposantonin | *D. wissmanniana* | Whole plant | (Cheng, Zhang et al. 2013) (wang, 2013) |
| 27 | Sesquiterpenes | isohyposantonin | *D. wissmanniana* | Whole plant | (Cheng, Zhang et al. 2013) |
| 28 | Sesquiterpenes | 5α-hydroxyasperilin | *D. wissmanniana* | Whole plant | (Cheng, Zhang et al. 2013) |
| 29 | Sesquiterpenes | 4α,15-dihydro-5α-hydroxyasperilin | *D. wissmanniana* | Whole plant | (Cheng, Zhang et al. 2013) |
| 30 | Sesquiterpenes | (5R,7R,10S)-4,5-epoxy-4,5-secoeudesma-1,3-dien-12,5-olide | *D. wissmanniana* | Whole plant | (Cheng, Zhang et al. 2013 |
| 31 | Sesquiterpenes | eudesma-3,11(13)-dien-12,5β-olide | *D. wissmanniana* | Whole plant | (Cheng, Zhang et al. 2013) |
| 32 | Sesquiterpenes | 3,4-dioxo-5α,7αH-3,4-secoeudesma-1,11(13)-dien-12-oic acid | *D. wissmanniana* | Whole plant | (Cheng, Zhang et al. 2013) |
| 33 | Sesquiterpenes | (2R,4E,6R,7S,9S,10R)-2-acetoxy-9-angeloyloxygermacra-4,11(13)-dien-12,6-olide | *D. wissmanniana* | Whole plant | (Cheng, Zhang et al. 2013) |
| 34 | Sesquiterpenes | 4E-9β-angeloyloxy-2α-hydroxy-7α,10αH-germacra-4,11(13)-dien-12,6α-olide | *D. wissmanniana* | Whole plant | (Cheng, Zhang et al. 2013) (wang, 2013) |
| 35 | Sesquiterpenes | (4E,6R,7S,9S,10R)-9-angeloyloxygermacra-4,11(13)-dien-12,6-olide | *D. wissmanniana* | Whole plant | (Cheng, Zhang et al. 2013) (wang, 2013) |
| 36 | Sesquiterpenes | 4E-9β-methacryloxy-7α,10αH-germacra-4,11(13)-dien-12,6α-olide | *D. wissmanniana* | Whole plant | (Cheng, Zhang et al. 2013) |
| 37 | Sesquiterpenes | 4E-9β-hydroxy-7α,10αH-germacra-4,11(13)-dien-12,6α-olide | *D. wissmanniana* | Whole plant | (Cheng, Zhang et al. 2013) |
| 38 | Sesquiterpenes | (1R,5S,7R,10R)-1-hydroxy-2-oxoeudesma-3,11(13)-dien-12-oic acid | *D. wissmanniana* | Whole plant | (Cheng, Zhang et al. 2013) |
| 39 | Sesquiterpenes | 4β,5α-dihydroxy-1-oxoeudesma-2,11(13)-dien-12,6β-olide | *D. wissmanniana* | Whole plant | (Cheng, Zhang et al. 2013) |
| 40 | Sesquiterpenes | 3-oxoeudesma-1,4,11(13)-trien-12,6β-olide | *D. wissmanniana* | Whole plant | (Cheng, Zhang et al. 2013) |
| 41 | Sesquiterpenes | (7R)-14(10→1)-abeoeudesma-1,3,5(10),11(13)-tetraen-12-oic acid | *D. wissmanniana* | Whole plant  Aerial part | (Cheng et al., 2013; wang, 2013) |
| 42 | Sesquiterpenes | (2S,7R,10R)-2-hydroxy-1-nor-3-oxoeudesm-4,11(13)-dien-12-oic acid | *D. wissmanniana* | Aerial part | (Cheng, Wang et al. 2014) |
| 43 | Sesquiterpenes | 14(10→1),15(4→2)-Abeo-7αH-eudesm-1,3,5(10),11(13)-tetraen-12-oic acid | *D. wissmanniana* | Aerial part | (wang, 2013; Cheng et al., 2014) |
| 44 | Sesquiterpenes | 1,10-dioxo-7αH-chromolaev-4,11(13)-dien-12-oic acid | *D. wissmanniana* | Aerial part | (Cheng, Wang et al. 2014) |
| 45 | Sesquiterpenes | 4β-hydroxy-5α,7αH-1-oxoeudesma-2,11(13)-dien-12-oic acid | *D. wissmanniana* | Aerial part | (Cheng, Wang et al. 2014) |
| 46 | Sesquiterpenes | 4α,5α-dihydroxy-7αH-1-oxoeudesma-2,11(13)-dien-12-oic acid | *D. wissmanniana* | Aerial part | (Cheng, Wang et al. 2014) |
| 47 | Sesquiterpenes | 1β-hydroxy-5α,7αH-eudesma-2,4(15),11(13)-trien-12-oic acid | *D. wissmanniana* | Aerial par | (Cheng, Wang et al. 2014 |
| 48 | Sesquiterpenes | 1α,5α-dihydroxy-7αH-eudesma-2,4(15),11(13)-trien-12-oic acid | *D. wissmanniana* | Aerial part | (Cheng, Wang et al. 2014) |
| 49 | Sesquiterpenes | 1β-hydroxy-7αH-eudesma-3,5,11(13)-trien-12-oic acid | *D. wissmanniana* | Aerial part | (Cheng, Wang et al. 2014) |
| 50 | Sesquiterpenes | 1α-hydroxy-5α,7αH-eudesma-2,4(15),11(13)-trien-12-oic acid | *D. wissmanniana* | Aerial part | (Cheng, Wang et al. 2014) |
| 51 | Sesquiterpenes | 1β-hydroxyilicic acid | *D. wissmanniana* | Aerial part | (wang, 2013; Cheng et al., 2014) |
| 52 | Sesquiterpenes | (4R,5R,6S,7S,9S,10R)-9-angeloyloxy-4,5-epoxygermacra-11(13)-en-12,6-olide | *D. wissmanniana* | Aerial part | (Cheng, Wang et al. 2014) |
| 53 | Sesquiterpenes | 4E-9β-angeloyloxy-7α,10α,11αH-germacra-4,11(13)-dien-12,6α-olide | *D. wissmanniana* | Aerial part | (Cheng, Wang et al. 2014) |
| 54 | Sesquiterpenes | winolides A | *D. wissmanniana* | Aerial part | (Cheng, Shao et al. 2014) |
| 55 | Sesquiterpenes | winolides B | *D. wissmanniana* | Aerial part | (Cheng, Shao et al. 2014) |
| 56 | Sesquiterpenes | winolides C | *D. wissmanniana* | Aerial part | (Cheng, Shao et al. 2014) |
| 57 | Sesquiterpenes | wissmannianlin C | *D. wissmanniana* | Aerial part | (wang, 2013) |
| 58 | Sesquiterpenes | wissmannianlin D | *D. wissmanniana* | Aerial part | (wang, 2013) |
| 59 | Sesquiterpenes | wissmannianlin F | *D. wissmanniana* | Aerial part | (wang, 2013) |
| 60 | Sesquiterpenes | wissmannianlin G | *D. wissmanniana* | Aerial part | (wang, 2013) |
| 61 | Sesquiterpenes | (1R,5S,7R,10R)-1-Hydroxy-2-oxo-isocostic acid | *D. wissmanniana* | Aerial part | (Cheng, 2012; wang, 2013) |
| 62 | Sesquiterpenes | hydroxy-7αH,8αH-eudesma-4,11 (13)-dien-8,12-olide | *D. wissmanniana* | Aerial part | (wang, 2013) |
| 63 | Sesquiterpenes | wissmannianlin I | *D. wissmanniana* | Aerial part | (wang, 2013) |
| 64 | Sesquiterpenes | wissmannianlin J | *D. wissmanniana* | Aerial part | (wang, 2013) |
| 65 | Sesquiterpenes | (2R,4E,6R,7S,9S,10S)-2-Acetoxy-9-angeloyloxy-germacra-4(5),11(13)-dien-12,6-olide | *D. wissmanniana* | Aerial part | (wang, 2013) |
| 66 | Sesquiterpenes | wissmannianlin K | *D. wissmanniana* | Aerial part | (wang, 2013) |
| 67 | Sesquiterpenes | wissmannianlin L | *D. wissmanniana* | Aerial part | (wang, 2013) |
| 68 | Sesquiterpenes | wissmannianlin B | *D. wissmanniana* | Whole plant | (Cheng, 2012) |
| 69 | Sesquiterpenes | chengwissmanolide A | *D. wissmanniana* | Whole plant | (Cheng, 2012) |
| 70 | Sesquiterpenes | chengwissmanolide B | *D. wissmanniana* | Whole plant | (Cheng, 2012) |
| 71 | Sesquiterpenes | chengwissmanolide C | *D. wissmanniana* | Whole plant | (Cheng, 2012) |
| 72 | Sesquiterpenes | ineupatolide D | *D. cappa* | Whole plant | (Wu, Tang et al. 2017) |
| 73 | Sesquiterpenes | ineupatolide E | *D. cappa* | Whole plant | (Wu, Tang et al. 2017) |
| 74 | Sesquiterpenes | dvaricin B | *D. cappa* | Whole plant  Aerial parts | (Wu, Tang et al. 2017；Wang, Li et al. 2012) |
| 75 | Sesquiterpenes | nepalolide C | *D. cappa* | Whole plant | (Wu, Tang et al. 2017) |
| 76 | Sesquiterpenes | inculacappolide | *D. cappa* | Whole plant | (Wu, Tang et al. 2017) |
| 77 | Sesquiterpenes | 2,3-epoxy-4-hydroxy-5-angeloxy-8-(2-methylbutyloxy)-germacra-11(13)-en-9-one-12,6-olide | *D. cappa* | Aerial parts | (Goswami, Baruah et al. 1984) |
| 78 | Sesquiterpenes | 2,3-epoxy-4-hydroxy-5-angeloxy-8-angelyl-germacra-11(13)-en-9-one-12,6-olide | *D. cappa* | Aerial parts | (Goswami, Baruah et al. 1984) |
| 79 | Sesquiterpenes | 2,4,9-trihydroxy-(5-methylbutyloxy)-8-angeloxy-germacra-11(13)-en-3-one-12,6-olide | *D. cappa* | Aerial parts | (Goswami, Baruah et al. 1984) |
| 80 | Sesquiterpenes | 2,4,9-trihydroxy-(5-angelyl)-8-angeloxy-germacra-11(13)-en-3-one-12,6-olide | *D. cappa* | Aerial parts | (Goswami, Baruah et al. 1984) |
| 81 | Sesquiterpenes | 4,8-dihydroxy-5-angeloxy-9-(2-  methylbutyl)-germacra-11(13)-en-3-one-12,6-olide | *D. cappa* | Aerial parts | (Goswami, Baruah et al. 1984) |
| 82 | Sesquiterpenes | 2,3-dihydroxy-9-angeloxy-germacra-4E, 11(13)-dien-12,6- olide | *D. cappa* | Aerial parts | (Goswami, Baruah et al. 1984) |
| 83 | Sesquiterpenes | β-farnesene | *D. cappa* | Aerial parts | (Bohlmann, Ahmed et al. 1982) |
| 84 | Sesquiterpenes | 1β,10α-epoxy-1,10-dihydrocaryophyllene | *D. cappa* | Aerial parts | (Bohlmann, Ahmed et al. 1982) |
| 85 | Sesquiterpenes | 2β,5-epoxy-5,10-dihydroxy-6α-angeloxy-9β-isobutyloxy-germacran-8α,12-olide | *D. cappa* | Aerial parts | (Wang, Li et al. 2012) |
| 86 | Sesquiterpenes | ineupatolide | *D. cappa*  D. eupatorioides | Aerial parts  Whole plant | (Wang, Li et al. 2012) (Baruah, Sharma et al. 1982) |
| 87 | Sesquiterpenes | inuviscolide | *D. cappa* | Aerial parts | (Wang, Li et al. 2012) |
| 88 | Sesquiterpenes | carabrone | *D. cappa*  *D. pterocaula* | Aerial parts  Whole plant | (Wang et al., 2012; Zhu et al., 2019) |
| 89 | Sesquiterpenes | ineupatorolide B | *D. pterocaula*  D. eupatorioides  *D. cappa* | Whole plant  Flowers | (Baruah et al., 1980; Yang et al., 2011; Zhu et al., 2019) |
| 90 | Sesquiterpenes | involucratolactone | *D. pterocaula* | Whole plant | (Zhu, Xiao et al. 2019) |
| 91 | Sesquiterpenes | 6α-hydroxy-4(15),10(14)-guaianadien-8β,12-olide | *D. pterocaula* | Whole plant | (Zhu, Xiao et al. 2019) |
| 92 | Sesquiterpenes | pterocaullin A | *D. pterocaula* | Whole plant | (Sheng-Lan, Chun-Li et al. 2019) |
| 93 | Sesquiterpenes | pterocaullin B | *D. pterocaula* | Whole plant | (Sheng-Lan, Chun-Li et al. 2019) |
| 94 | Sesquiterpenes | pterocaullin C | *D. pterocaula* | Whole plant | (Sheng-Lan, Chun-Li et al. 2019) |
| 95 | Sesquiterpenes | pterocaullin D | *D. pterocaula* | Whole plant | (Sheng-Lan, Chun-Li et al. 2019) |
| 96 | Sesquiterpenes | hierapolitanin A | *D. pterocaula* | Whole plant | (Sheng-Lan, Chun-Li et al. 2019) |
| 97 | Sesquiterpenes | onopordopicrin | *D. pterocaula* | Whole plant | (Sheng-Lan, Chun-Li et al. 2019) |
| 98 | Sesquiterpenes | 1R, 4S, 5R, 6R, 7R, 8S, 10R-methyl 1,6-dihydroxy-8-ethacryloxyeudesm-11(13)-en-15-oic acid-12-oate | *D. pterocaula* | Whole plant | (Sheng-Lan, Chun-Li et al. 2019) |
| 99 | Sesquiterpenes | 3α-(2,3-epoxy-2-methylbutyryloxy)-4α-hydroxy-11-hydroperoxy-eudesm-6-en-8-one | *D. pterocaula* | Whole plant | (Sheng-Lan, Chun-Li et al. 2019) |
| 100 | Sesquiterpenes | ineupatoriol | D. eupatorioides | Whole plant | (Baruah et al., 1982) |
| 101 | Sesquiterpenes | ineupatorolide A | D. eupatorioides | Whole plant | (Baruah et al., 1980) |
| 102 | Sesquiterpenes | ineupatolide A | *D. cappa* | Aerial parts | (Wang et al., 2012) |
| 103 | Sesquiterpenes | ineupatolide B | *D. cappa* | Aerial parts | (Wang et al., 2012) |
| 104 | Sesquiterpenes | ineupatolide C | *D. cappa* | Aerial parts | (Wang et al., 2012) |
| 105 | Sesquiterpenes | bigelovin | *D. nervosa* | Aerial parts  Whole plant | (LAN, 2010; Yan LAN Jin Huizi, 2011) |
| 106 | Sesquiterpenes | ophiopogonoside A | *D. cappa* | Whole plant | (Zhou, 2017) |
| 107 | Sesquiterpenes | loliolide | *D. wissmanniana*  *D. cappa* | Whole plant  Aerial parts | (LAN, 2010; Yan LAN Jin Huizi, 2011; Cheng, 2012) |
| 108 | Sesquiterpenes | dehydrololiolide | *D. wissmanniana* | Whole plant | (Cheng, 2012) |
| 109 | Sesquiterpenes | dehydrovomifoliol | *D. wissmanniana* | Whole plant | (Cheng, 2012) |
| 110 | Sesquiterpenes | dihydroactinidiolide | *D. pterocaula* | Whole plan | Chun-Li et al. 2019) |
| 111 | diterpenes | maoxiucaioside A | *D. wissmanniana* | Aerial parts | (Li et al., 2023) |
| 112 | diterpenes | (13E)-neoderoda-3,15-diene-15,18-diol | *D. nervosa* | Aerial parts  Whole plant | (LAN, 2010; Yan et al., 2011) |
| 113 | diterpenes | vanclevic acid B methyl ester | *D. nervosa* | Aerial parts  Whole plant | (LAN, 2010; Yan et al., 2011) |
| 114 | diterpenes | butanedioic acid, [1,3,4,7,8,8ahexahydro-1-(5-hydroxy-3- methyl-3-pentenyl)-  1,2,5-trimethyl-4a(2H)-naphthalenyl] methyl ester | *D. nervosa* | Whole plant | (LAN, 2010) |
| 115 | diterpenes | nervoyan A | *D. nervosa* | Whole plant | (LAN, 2010) |
| 116 | diterpenes | nervoyan B | *D. nervosa* | Whole plant | (LAN, 2010) |
| 117 | diterpenes | nervoyan C | *D. nervosa* | Whole plant | (LAN, 2010) |
| 118 | diterpenes | cis-abienol | *D. cappa* | Root | (Wu et al., 2010) |
| 119 | diterpenes | 12－demethylmulticauline | *D. cappa* | Root | (Li et al., 2020) |
| 120 | triterpenes | sterculin A | *D. nervosa* | aerial parts | (Li et al., 2023) |
| 121 | triterpenes | shionone | *D. nervosa* | Whole plant  Aerial parts | (LAN, 2010; Yan LAN Jin Huizi, 2011) |
| 122 | triterpenes | ursolic acid | *D. nervosa*  *D. pterocaula* | Whole plant  Aerial parts  Root | (Xie et al., 2007a; LAN, 2010; Yan LAN Jin Huizi, 2011; Tai Zhigang, 2014) |
| 123 | triterpenes | 3β-hydroxy-20-taraxasten-22-one palmitate | *D. cappa* | Flowers | (Wu et al., 2014) |
| 124 | triterpenes | taraxasterol palmitate | *D. cappa* | Flowers | (Wu et al., 2014) |
| 125 | triterpenes | squalene | *D. cappa* | Aerial parts | (Bohlmann et al., 1982) |
| 126 | triterpenes | darma-20,24-dien-3β-O-acetate | *D. cappa* | Aerial parts | (Zheng et al., 2015) |
| 127 | triterpenes | darma -20,24-dien-3β-ol | *D. cappa* | Aerial parts | (Zheng et al., 2015) |
| 128 | triterpenes | epirfiedelanol | *D. cappa* | Aerial parts  Root | (Wu et al., 2010; Zheng et al., 2015) |
| 129 | triterpenes | friedelin | *D. cappa* | Aerial parts  Root  Flowers  Whol plant | (Xie et al., 2007a; Wu et al., 2010; Yang et al., 2011; Zheng et al., 2015; Zhou, 2017) |
| 130 | triterpenes | β-amyrin | *D. pterocaula*  *D. cappa* | Root  Whole plan | (Xie et al., 2007a; Tai Zhigang, 2014; Zhou, 2017) |
| 131 | triterpenes | erythordiol | *D. pterocaula* | Root | (Tai Zhigang, 2014) |
| 132 | triterpenes | oleanolic acid | *D. pterocaula*  *D. cappa* | Root  Whole plant | (Xie et al., 2007a; Tai Zhigang, 2014) |
| 133 | triterpenes | 2β,3β,23α-trihyroxyolean-12-en-28-acid | *D. pterocaula* | Root | (Tai Zhigang, 2014) |
| 134 | triterpenes | oleanoic acid 28-O-β-D -glucopyranosyl ester | *D. pterocaula* | Root | (Tai Zhigang, 2014) |
| 135 | triterpenes | oleanoic acid 3-O-β-D -glucopyranoside | *D. pterocaula* | Root | (Tai Zhigang, 2014) |
| 136 | triterpenes | oleanoic acid 3-O-(β-D -glucopyranosyl)-28-O-β-D -glucopyranosyl ester | *D. pterocaula* | Root | (Tai Zhigang, 2014) |
| 137 | triterpenes | 2β-hydroxyolean-3-O-(β-D-glucopyranosyloxy)-12-en-23，28-dioicacid | *D. pterocaula* | Root | (Tai Zhigang, 2014) |
| 138 | triterpenes | 3-O-β-glucopyranosyl-(1→4)-β-D-glucuronopyranoside-23-hydroxyolean-12-en-28-oic acid 28-O-β-D -glucopyranosyl ester | *D. pterocaula* | Root | (Tai Zhigang, 2014) |
| 139 | triterpenes | 3-O-β-D-glucopyranosyl pomolic acid-28-O-β-D -glucopyranosylester | *D. pterocaula* | Root | (Tai Zhigang, 2014) |
| 140 | triterpenes | 3-O-α-L-arabinpyranosyl-20，19，24-trihydroxy ursolic acid | *D. pterocaula* | Root | (Tai Zhigang, 2014) |
| 141 | triterpenes | [(3β)-Dammara-20,24-dien-3-ylacetate](https://www.chemsrc.com/en/cas/52914-31-5_218892.html) | *D. nervosa* | Root | (Duoging et al., 1997) |
| 142 | triterpenes | lupeol acetate | *D. cappa* | Flowers | (Yang et al., 2011) |
| 143 | triterpenes | epifriedelanol | *D. cappa* | Whole plant | (Guo Qilei, 2007; Xie et al., 2007a; Zhou, 2017) |
| 144 | triterpenes | α-amyrin | *D. cappa* | Whole plant | (Xie et al., 2007a; Zhou, 2017) |
| 145 | inositol angelates | 4-Acetate-1,2,3,5-tetrakis(2-methyl-2-butenoate) inositol | *D. wissmanniana* | Aerial part | (wang, 2013; Wang et al., 2013) |
| 146 | inositol angelates | 3-Acetate-1,2,4,5-tetrakis(2-methyl-2-butenoate) inositol | *D. wissmanniana* | Aerial part | (wang, 2013; Wang et al., 2013) |
| 147 | inositol angelates | myoinositol-1,4,5-triangelate | *D. cappa* | Whole plant | (Wu et al., 2015) |
| 148 | inositol angelates | myoinositol-2-acetate-1,4,5-triagelate | *D. cappa* | Whole plant | (Wu et al., 2015) |
| 149 | inositol angelates | myoinositol-3-acetate-1,4,5-triangelate | *D. cappa* | Whole plant | (Wu et al., 2015) |
| 150 | inositol angelates | myoinositol-2-acetate-3,5,6-triangelate | *D. cappa* | Whole plant | (Wu et al., 2015) |
| 151 | inositol angelates | myoinositol-2-isobutyryloxy-1,4,5-triangelate | *D. cappa* | Whole plant | (Wu et al., 2015) |
| 152 | inositol angelates | myoinositol-1-(4-methylsenecioyloxy)-4,5-diangelate | *D. cappa* | Whole plant | (Wu et al., 2015) |
| 153 | inositol angelates | myoinositol-1,3,4,6-tetraangelate | *D. cappa* | Whole plant | (Bohlmann et al., 1982; Wu et al., 2015) |
| 154 | inositol angelates | myoinositol-2,4,5,6-tetraangelate | *D. cappa* | Whole plant | (Bohlmann et al., 1982; Wu et al., 2015) |
| 155 | inositol angelates | cis-1,2,3,5-trans-4,6-inositol-2,3,6-triangelate | *D. cappa* | Whole plant | (Zou et al., 2008; Cheng, 2012; Wu et al., 2015) |
| 156 | inositol angelates | l-inositol-2,3,6-triangelate | *D. cappa* | Whole plant | (Wu et al., 2015) |
| 157 | inositol angelates | l-inositol-1,3,4-triangelate | *D. cappa* | Whole plant | (Wu et al., 2015) |
| 158 | inositol angelates | l-inositol-2-acetate-3,4,6-triangelate | *D. cappa* | Whole plant | (Wu et al., 2015) |
| 159 | inositol angelates | l-inositol-1,2-diacetate-3,4,6-triangelate | *D. cappa* | Whole plant | (Wu et al., 2015) |
| 160 | inositol angelates | l-inositol-3-(4-methylsenecioyloxy)-1,6-diangelate | *D. cappa* | Whole plant | (Wu et al., 2015) |
| 161 | inositol angelates | l-inositol-6-(4-methylsenecioyloxy)-2,3,4-triangelate | *D. cappa* | Whole plant | (Wu et al., 2015) |
| 162 | inositol angelates | l-inositol-1,2,3,5-tetraangelate | *D. cappa* | Whole plant | (Zou et al., 2008; Wu et al., 2015) |
| 163 | inositol angelates | l-inositol-2,3,5,6-tetraangelate | *D. cappa* | Whole plant | (Bohlmann et al., 1982; Wu et al., 2015) |
| 164 | Phenylpropanoids | C-Veratroylglycol | *D. wissmanniana* | Aerial part | (wang, 2013) |
| 165 | Phenylpropanoids | 2,3-Dihydroxy-1-(4-hydroxy-3,5-dimethoxyphenyl)-1-propanone | *D. wissmanniana* | Aerial part | (wang, 2013) |
| 166 | Phenylpropanoids | Caffeic acid | *D. wissmanniana* | Aerial part | (wang, 2013) |
| 167 | Phenylpropanoids | trans-p-hydroxycinnamic acid | *D. wissmanniana* | Aerial part | (wang, 2013) |
| 168 | Phenylpropanoids | 3,5-di-O-caffeoyl quinic acid | *D. wissmanniana* | Aerial part | (wang, 2013; Huan-yu et al., 2014) |
| 169 | Phenylpropanoids | 4,5-di-O-caffeoyl quinic acid | *D. wissmanniana* | Aerial part | (wang, 2013) |
| 170 | Phenylpropanoids | 3,4-di-O-caffeoyl quinic acid | *D. wissmanniana* | Aerial part | (wang, 2013; Huan-yu et al., 2014) |
| 171 | Phenylpropanoids | scopoletin | *D. wissmanniana*  *D. cappa* | Whole plant  Aerial parts | (Xie et al., 2007b; Cheng, 2012; Zheng et al., 2015; Zhou, 2017) |
| 172 | Phenylpropanoids | nervolans A | *D. nervosa* | Aerial parts | (LAN, 2010; Yan et al., 2010) |
| 173 | Phenylpropanoids | nervolans B | *D. nervosa* | Aerial parts | (LAN, 2010; Yan et al., 2010) |
| 174 | Phenylpropanoids | nervolans C | *D. nervosa* | Aerial parts | (LAN, 2010; Yan et al., 2010) |
| 175 | Phenylpropanoids | coniferyl diangelate | *D. nervosa* | Aerial parts | (LAN, 2010; Yan et al., 2010) |
| 176 | Phenylpropanoids | sinapyl diangelate | *D. nervosa* | Aerial parts | (LAN, 2010; Yan et al., 2010) |
| 177 | Phenylpropanoids | 3-Hydroxy-1-(4-hydroxy-3,5-dimethoxyphenyl)-1-propanone | *D. nervosa* | Whole plant | (LAN, 2010) |
| 178 | Phenylpropanoids | ω-Hydroxypropioguaiacone | *D. nervosa* | Whole plant | (LAN, 2010) |
| 179 | Phenylpropanoids | coniferaldehyde | *D. nervosa* | Whole plant | (LAN, 2010; Wu et al., 2010; Zhu et al., 2019) |
| 180 | Phenylpropanoids | dihydrosyringin | *D. nervosa* | Whole plant | (LAN, 2010) |
| 181 | Phenylpropanoids | 1,5-di-O-caffeoylquinic acid | *D. cappa* | Whole plant | (Huan-yu et al., 2014) |
| 182 | Phenylpropanoids | 1,3,5-tri-0-caffeoylquinic acid | *D. cappa* | Whole plant | (Huan-yu et al., 2014) |
| 183 | Phenylpropanoids | methyl3,5-di-O-caffeoyl- quinate | *D. cappa* | Whole plant | (Huan-yu et al., 2014) |
| 184 | Phenylpropanoids | methyl3,4-di-O-caffeoy lquinate | *D. cappa* | Whole plant | (Huan-yu et al., 2014) |
| 185 | Phenylpropanoids | Ethyl3,4-di-O-caffeoylquinate | *D. cappa* | Whole plant | (Huan-yu et al., 2014) |
| 186 | Phenylpropanoids | ethyl4,5-di- 0-caffcoylquinate | *D. cappa* | Whole plant | (Huan-yu et al., 2014) |
| 187 | Phenylpropanoids | 2,6,2′,6′-tetramethoxy-4,4′-bis(2,3-epoxy-1-hydroxypropyl) biphenyl | *D. pterocaula* | Whole plant | (Zhu et al., 2019) |
| 188 | Phenylpropanoids | pinoresinol | *D. pterocaula* | Whole plant | (Sheng-Lan et al., 2019) |
| 189 | Phenylpropanoids | syringaresinol | *D. pterocaula* | Whole plant | (Sheng-Lan et al., 2019) |
| 190 | Phenylpropanoids | isoscopoletin | *D. nervosa*  *D. cappa*  *D. pterocaula* | Whole plant  Aerial parts | (LAN, 2010; Zheng et al., 2015; Sheng-Lan et al., 2019) |
| 191 | Phenylpropanoids | sinapylaldehyde | *D. cappa* | Root | (Wu et al., 2010) |
| 192 | Phenylpropanoids | p-coumaric acid | *D. cappa* | Root | (Wu et al., 2010) |
| 193 | Phenylpropanoids | chlorogenic acid | *D. cappa* | Root | (Wu et al., 2010) |
| 194 | Phenylpropanoids | macranthoin F | *D. cappa* | Root | (Wu et al., 2010) |
| 195 | Phenylpropanoids | macranthoin G | *D. cappa* | Root | (Wu et al., 2010) |
| 196 | Phenylpropanoids | methyl-4,5-dicaffeoylquinate | *D. cappa* | Root | (Wu et al., 2010) |
| 197 | Phenylpropanoids | scopolin | *D. cappa* | Root  Aerial parts | (Guo et al., 2007; Zheng et al., 2015) |
| 198 | Phenylpropanoids | 4-allyl-2,6-dimethoxyphenol-1-O-β-glucopyranoside | *D. cappa* | Root | (Wu, Shan et al. 2010) |
| 199 | Phenylpropanoids | 4-[(6-O-(E)-caffeoyl)-β-D-glucopyranosyl] vanillic acid | *D. cappa* | Root | (Wu, Shen et al. 2013) |
| 200 | Phenylpropanoids | coniferyl aldehyde | *D. cappa* | Whole plant | (Xie, Zhang et al. 2007) |
| 201 | Phenylpropanoids | caffeic acid ethyl ester | *D. cappa* | Root | (Lincai et al., 2017) |
| 202 | Phenylpropanoids | 3-(4-hydroxy-3-methoxyphenyl)-propenal | *D. cappa* | Root | (Lincai et al., 2017) |
| 203 | Flavonoids | 23-o-Acetylsilychristin A | *D. wissmanniana* | Aerial part | (wang, 2013; Wang et al., 2013) |
| 204 | Flavonoids | silychristin A | *D. wissmanniana* | Aerial part | (wang, 2013; Wang et al., 2013) |
| 205 | Flavonoids | silychristin B | *D. wissmanniana* | Aerial part | (wang, 2013; Wang et al., 2013) |
| 206 | Flavonoids | isosilychristin | *D. wissmanniana* | Aerial part | (wang, 2013; Wang et al., 2013) |
| 207 | Flavonoids | isohydnocarpin | *D. wissmanniana* | Aerial part | (wang, 2013; Wang et al., 2013) |
| 208 | Flavonoids | 2,3-Dehydrosilychristin | *D. wissmanniana* | Aerial part | (wang, 2013; Wang et al., 2013) |
| 209 | Flavonoids | silybin A | *D. wissmanniana* | Aerial part | (wang, 2013; Wang et al., 2013) |
| 210 | Flavonoids | silybin B | *D. wissmanniana* | Aerial part | (wang, 2013; Wang et al., 2013) |
| 211 | Flavonoids | isosilybin A | *D. wissmanniana* | Aerial part | (wang, 2013; Wang et al., 2013) |
| 212 | Flavonoids | hydnocarpin | *D. wissmanniana* | Aerial part | (wang, 2013; Wang et al., 2013) |
| 213 | Flavonoids | silydianin | *D. wissmanniana* | Aerial part | (wang, 2013; Wang et al., 2013) |
| 214 | Flavonoids | luteolin | *D. wissmanniana*  *D. cappa* | Aerial part  Whole plant  Flowers | (Xie et al., 2007b; Yang et al., 2011; Cheng, 2012; wang, 2013; Zheng et al., 2015; Zhou, 2017) |
| 215 | Flavonoids | apigenin | *D. wissmanniana*  *D. cappa* | Aerial part  Root  Flowers  Whole plant | (Xie et al., 2007b; Wu et al., 2010; Yang et al., 2011; wang, 2013; Zheng et al., 2015; Lincai et al., 2017) |
| 216 | Flavonoids | chrysoerio | *D. wissmanniana* | Aerial part | (wang, 2013; Zheng et al., 2015) |
| 217 | Flavonoids | 3-O-Methylquercetin | *D. wissmanniana* | Aerial part | (wang, 2013) |
| 218 | Flavonoids | 4',5,6-Trihydroxy-3,7-dimethoxyflavone | *D. wissmanniana* | Aerial part | (wang, 2013) |
| 219 | Flavonoids | 4',5-Dihydroxy-3,3',7,8-tetramethoxyflavone | *D. wissmanniana* | Aerial part | (wang, 2013) |
| 220 | Flavonoids | artemetin | *D. wissmanniana* | Aerial part | (wang, 2013; Zheng et al., 2015) |
| 221 | Flavonoids | 2',5,5-Dihydroxy-3,4',6,7-tetramethoxyflavone | *D. wissmanniana* | Aerial part | (wang, 2013) |
| 222 | Flavonoids | (2R,3R)- (+)-Taxifolin | *D. wissmanniana* | Aerial part | (wang, 2013) |
| 223 | Flavonoids | Dihydrokaempferol | *D. wissmanniana* | Aerial part | (wang, 2013) |
| 224 | Flavonoids | Brickellin | *D. wissmanniana* | Whole plant | (Cheng, 2012) |
| 225 | Flavonoids | 5-O-Demethylapulein | *D. wissmanniana* | Whole plant | (Cheng, 2012) |
| 226 | Flavonoids | 5-Hydroxy-3,6,7,3',4'-pentamethoxyflavone | *D. wissmanniana* | Whole plant | (Cheng, 2012) |
| 227 | Flavonoids | Penduletin | *D. wissmanniana* | Whole plant | (Cheng, 2012) |
| 228 | Flavonoids | chrysosptertin B | *D. wissmanniana* | Whole plant | (Cheng, 2012) |
| 229 | Flavonoids | silychristin A | *D. wissmanniana* | Whole plant | (Cheng, 2012) |
| 230 | Flavonoids | silychristin B | *D. wissmanniana* | Whole plant | (Cheng, 2012) |
| 231 | Flavonoids | silybin A | *D. wissmanniana* | Whole plant | (Cheng, 2012) |
| 232 | Flavonoids | silybin B | *D. wissmanniana* | Whole plant | (Cheng, 2012) |
| 233 | Flavonoids | daidzein | *D. nervosa* | Aerial parts | (Li et al., 2023) |
| 234 | Flavonoids | 5-hydroxy-7,4′-dimethoxyflavone | *D. nervosa* | Aerial parts | (Li et al., 2023) |
| 235 | Flavonoids | kaempferol | *D. nervosa* | Whole plant  Aerial parts | (LAN, 2010; Yan LAN Jin Huizi, 2011) |
| 236 | Flavonoids | spinacetin | *D. nervosa* | Whole plant  Aerial parts | (LAN, 2010; Yan LAN Jin Huizi, 2011) |
| 237 | Flavonoids | acacetin | *D. nervosa* | Whole plant | (LAN, 2010) |
| 238 | Flavonoids | rutin | *D. nervosa* | Whole plant | (LAN, 2010) |
| 239 | Flavonoids | (2R,3R)-5′-methoxy-3,5,7,2′-tetrahydroxyflavanone | *D. cappa* | Aerial parts | (Baruah et al., 1979) |
| 240 | Flavonoids | (2S)-5,7,2′,5′-tetrahydroxyflavanone | *D. cappa* | Aerial parts | (Baruah et al., 1979) |
| 241 | Flavonoids | 7,5′-dimethoxy-3,5,2′-trihydroxyflavone | *D. cappa* | Aerial parts | (Baruah et al., 1979) |
| 242 | Flavonoids | 2',5-dihydroxy-3,6,7,4',5'-pentamethoxyflavone | *D. cappa* | Aerial parts | (Zheng et al., 2015) |
| 243 | Flavonoids | chrysosplenol C | *D. cappa* | Aerial parts | (Zheng et al., 2015) |
| 244 | Flavonoids | apigenin-5-O-β-D-glucopyranoside | *D. cappa* | Aerial parts | (Zheng et al., 2015) |
| 245 | Flavonoids | luteolin-3-methyl, luteolin-3-methylether-4'-O-β-D-glucopyranoside | *D. cappa* | Aerial parts | (Zheng et al., 2015) |
| 246 | Flavonoids | luteolin-4'-O-β-D-glucopyranoside | *D. cappa* | Aerial parts | (Zheng et al., 2015) |
| 247 | Flavonoids | luteolin-3'-O-β-D-glucopyranoside | *D. cappa* | Root | (Wu et al., 2010) |
| 248 | Flavonoids | fortuneletin | *D. cappa* | Flowers | (Yang et al., 2011) |
| 249 | Flavonoids | luteolin 4′-methyl ether | *D. cappa* | Flowers | (Yang et al., 2011) |
| 250 | Flavonoids | luteolin-7-O-β-D-glucuronide ethyl ester | *D. cappa* | Whole plant | (Zhou, 2017) |
| 251 | Flavonoids | apigenin-7-O-β-D-glucoside | *D. cappa* | Whole plant | (Zhou, 2017) |
| 252 | Flavonoids | luteolin-7-O-β-D-rutinoside | *D. cappa* | Whole plant | (Zhou, 2017) |
| 253 | Flavonoids | hydnocarpin-D | *D. cappa* | Whole plant | (Zhou, 2017) |
| 354 | Flavonoids | luteolin-7-O-β-D-glucoside | *D. cappa* | Whole plant | (Zhou, 2017) |
| 255 | Flavonoids | luteolin-4'-O-β-D-glucoside | *D. cappa* | Whole plant | (Zhou, 2017) |
| 256 | Flavonoids | quercetin-3-O-β-D-glucoside | *D. cappa* | Whole plant | (Zhou, 2017) |
| 257 | Flavonoids | quercitrin | *D. pterocaula* | Whole plant | (Tai Zhigang, 2013) |
| 258 | Flavonoids | 5-hydroxy-7,3',4'-trimethoxy-flavone | *D. pterocaula* | Whole plant | (Tai Zhigang, 2013) |
| 259 | Flavonoids | isoquercitrin | *D. pterocaula* | Whole plant | (Tai Zhigang, 2013) |
| 260 | Flavonoids | terniflorin | *D. pterocaula* | Whole plant | (Tai Zhigang, 2013) |
| 261 | Flavonoids | apigenin-7-O-β-D-glucopyranosyl-(1'''→2'')-β-D-glucopyranosid | *D. pterocaula* | Whole plant | (Tai Zhigang, 2013) |
| 262 | Flavonoids | Pigenin-7-O-(6″-feruloyl)-β-D-glucopyranoside | *D. pterocaula* | Whole plant | (Tai Zhigang, 2013) |
| 263 | Flavonoids | vitexicarpin | *D. cappa* | Root | (Li et al., 2020) |
| 264 | Steroids | *β*-Sitosterol | *D. nervosa*  *D. pterocaula*  *D. cappa*  *D. wissmanniana* | Whole plant  Aerial parts  Root | (Guo Qilei, 2007; Xie et al., 2007a; LAN, 2010; Wu et al., 2010; Yan LAN Jin Huizi, 2011; Tai Zhigang, 2013; wang, 2013; Lincai et al., 2017) |
| 265 | Steroids | daucosterol | *D. nervosa*  *D. cappa*  *D. pterocaula* | Whole plant  Aerial parts  Root | (Duoging et al., 1997; Guo Qilei, 2007; Xie et al., 2007a; LAN, 2010; Wu et al., 2010; Yan LAN Jin Huizi, 2011; Tai Zhigang, 2013; Lincai et al., 2017) |
| 266 | Steroids | *α*-Spinasterol | *D. nervosa* | Whole plant  Aerial parts | (LAN, 2010; Yan LAN Jin Huizi, 2011) |
| 267 | Steroids | *α*-Spinasterol-3-*O*-*β*-*D*-glucoside | *D. nervosa* | Whole plant  Aerial parts | (LAN, 2010)  (Yan LAN Jin Huizi, 2011) |
| 268 | Steroids | 24S-Ethyl-5α-cholesta-7,22E-dien-3α-ol-β-galactopyranoside | *D. nervosa* | Whole plant  Aerial parts | (LAN, 2010; Yan LAN Jin Huizi, 2011) |
| 269 | Steroids | ergosterol endoperoxide | *D. nervosa* | Aerial parts | (Li et al., 2023) |
| 270 | Steroids | gramistereol | *D. nervosa* | Aerial parts | (Li et al., 2023) |
| 271 | Steroids | 7-oxo-β-sitosterol | *D. nervosa*  *D. cappa* | Aerial parts  Whole plant | (Xie et al., 2007a; Li et al., 2023) |
| 272 | Steroids | 7-oxo-3β-stigmasterol | *D. nervosa* | Aerial parts | (Li et al., 2023) |
| 273 | Steroids | 6β-hydroxy-stigmasta-4,22-dien-3-one | *D. nervosa* | Aerial parts | (Li et al., 2023) |
| 274 | Steroids | stigmasterol | *D. nervosa*  *D. cappa* | Aerial parts  Root  Flowers  Whole plant | (Duoging et al., 1997; Xie et al., 2007a; Yang et al., 2011; Zheng et al., 2015; Li et al., 2023) |
| 275 | Steroids | β-stigmasterol-3-O-β-D-glucopyranoside | *D. nervosa* | Aerial parts | (Li et al., 2023) |
| 276 | Steroids | dammara-20,24-dien-3β-O-acetate | *D. cappa* | Root | (Wu et al., 2010) |
| 277 | Steroids | dammara-20,24-dien-3β-ol | *D. cappa* | Root | (Wu et al., 2010) |
| 278 | Steroids | stigmast-4-en-3-one | *D. cappa* | Whole plant | (Xie et al., 2007a) |
| 279 | Steroids | stigmasta-4,22-dien-3-one | *D. cappa* | Whole plant | (Xie et al., 2007a) |
| 280 | Steroids | stigmast-5-ene-3β,7β-diol | *D. cappa* | Whole plant | (Xie et al., 2007a) |
| 281 | Steroids | stigmasta-5,22-diene-3β,7β-diol | *D. cappa* | Whole plant | (Xie et al., 2007a) |
| 282 | Steroids | stigmast-5-ene-3β,7α-diol | *D. cappa* | Whole plant | (Xie et al., 2007a) |
| 283 | Steroids | stigmasta-5,22-diene-3β,7α-diol | *D. cappa* | Whole plant | (Xie et al., 2007a) |
| 284 | Steroids | stigmasta-5,22-dien-3β-O-7-one | *D. cappa* | Aerial parts | (Zheng et al., 2015) |
| 285 | Steroids | damane － 20，24 － diene － 3β － acetyl | *D. cappa* | Root | (Li et al., 2020) |
| 286 | Fatty acids | 1-glyceryl linoleate | *D. nervosa* | Root | (Duoging et al., 1997) |
| 287 | Fatty acids | 1,3-dioleate glyceride | *D. nervosa* | Root | (Duoging et al., 1997) |
| 288 | Fatty acids | plamitic acid | *D. nervosa* | Whole plant | (LAN, 2010) |
| 289 | Fatty acids | docosanoic acid | *D. cappa* | Root | (Wu et al., 2010) |
| 290 | Fatty acids | linoleic acid | *D. cappa* | Root  Aerial parts | (Wu et al., 2010; Zheng et al., 2015) |
| 291 | Fatty acids | azelaic acid | *D. cappa* | Whole plant | (Xie et al., 2007b) |
| 292 | Fatty acids | dotriacontanic acid | *D. cappa* | Whole plant | (Xie et al., 2007b) |
| 293 | Fatty acids | octacosanoic acid | *D. cappa* | Root | (Guo et al., 2007; Guo Qilei, 2007) |
| 294 | Fatty acids | palmitic acid | *D. cappa* | Root  Aerial parts | (Zheng et al., 2015; Lincai et al., 2017) |
| 295 | Fatty acids | linoleic acid methyl ester | *D. cappa* | Aerial parts | (Zheng et al., 2015) |
| 296 | Fatty acids | (E)-9,12,13-trihydroxyoetadee-10-enoie acid | *D. cappa* | Aerial parts | (Zheng et al., 2015) |
| 297 | Other | N-(2-aminophenyl) urea | *D. wissmanniana* | Aerial parts | (wang, 2013) |
| 298 | Other | Copranosyloxyjasmonic acid | *D. wissmanniana* | Aerial parts | (wang, 2013) |
| 299 | Other | 4-(3-Hydroxybutyl)-3-methylenetetrahydro-2H-pyran-2-one | *D. wissmanniana* | Whole plant | (Cheng, 2012) |
| 300 | Other | Avicequinone D | *D. wissmanniana* | Whole plant | (Cheng, 2012) |
| 301 | Other | 3-Formyl-indole | *D. wissmanniana* | Whole plant | (Cheng, 2012) |
| 302 | Other | 3-(Hydroxyl-acetyl)-1H-indole | *D. wissmanniana* | Whole plant | (Cheng, 2012) |
| 303 | Other | N-(2-Aminophenyl) urea | *D. wissmanniana* | Whole plant | (Cheng, 2012) |
| 304 | Other | Vanillyl alcohol | *D. wissmanniana* | Whole plant | (Cheng, 2012) |
| 305 | Other | 2-Octenol | *D. nervosa* | Whole plant | (LAN, 2010) |
| 306 | Other | Salicylaldehyde | *D. nervosa* | Whole plant | (LAN, 2010) |
| 307 | Other | 4-Hydroxybenzaldehyde | *D. nervosa* | Whole plant | (LAN, 2010) |
| 308 | Other | 1,3,5-trimethoxybenzene | *D. nervosa* | Whole plant | (LAN, 2010) |
| 309 | Other | benzyl-β-D-glucopyranoside | *D. nervosa* | Whole plant  Aerial parts | (LAN, 2010; Yan LAN Jin Huizi, 2011) |
| 310 | Other | 2-Phenylethyl-β-D-glucopyranoside | *D. nervosa* | Whole plant  Aerial parts | (LAN, 2010; Yan LAN Jin Huizi, 2011) |
| 311 | Other | vanillic acid | *D. nervosa* | Whole plant  Root | (Guo Qilei, 2007; LAN, 2010; Wu et al., 2010) |
| 312 | Other | isovanilin | *D. nervosa* | Whole plant | (LAN, 2010) |
| 313 | Other | (Z)-3-Hexenyl-β-D-glucopyranoside | *D. nervosa* | Whole plant | (LAN, 2010) |
| 314 | Other | boscialin 4'-O-glucoside | *D. nervosa* | Whole plant | (LAN, 2010) |
| 315 | Other | vanillin | *D. cappa*  *D. wissmanniana* | Root  Whole plant  Aerial part | (Xie et al., 2007b; Wu et al., 2010)  (王春辉 2013) |
| 316 | Other | protocatechualdehyde | *D. cappa* | Root | (Wu et al., 2010) |
| 317 | Other | 4-hydroxybenzoic acid | *D. cappa*  *D. wissmanniana* | Root  Aerial part | (Wu et al., 2010)  (王春辉 2013) |
| 318 | Other | syringic acid | *D. cappa* | Root  Whole plant | (Xie et al., 2007b; Wu et al., 2010) |
| 319 | Other | vanillic acid-4-O-β-glucopyranoside | *D. cappa* | Root | (Wu et al., 2010) |
| 320 | Other | tachioside | *D. cappa* | Root | (Wu et al., 2010) |
| 321 | Other | isotachioside | *D. cappa* | Root | (Wu et al., 2010) |
| 322 | Other | osmantolide | *D. cappa* | Root | (Wu et al., 2010) |
| 323 | Other | 3-methyl-2-thiophenecarboxylic acid | *D. cappa* | Root | (Wu et al., 2010) |
| 324 | Other | 5-hydroxymethylfurfural | *D. cappa* | Root | (Wu et al., 2010) |
| 325 | Other | ceplignan | *D. cappa* | Root | (Wu et al., 2010) |
| 326 | Other | cleomiscosin C | *D. cappa* | Flower | (Yang et al., 2011) |
| 327 | Other | 3,4-dihydroxy-benzoic acid | *D. cappa* | Flower | (Yang et al., 2011) |
| 328 | Other | 3-O-[β-D-apiofurarnosyl-(1-6)-β-D-glucopyranoxy]-6-hydroxy-p-cymene | *D. cappa* | Root | (Wu et al., 2013) |
| 329 | Other | aurantiamide acetate | *D. cappa* | Whole plant | (Xie et al., 2007b) |
| 330 | Other | aurantiamide benzoate | *D. cappa* | Whole plant | (Xie et al., 2007b) |
| 331 | Other | physcion | *D. cappa* | Whole plant | (Xie et al., 2007b) |
| 332 | Other | syringaldehyde | *D. cappa*  *D. pterocaula* | Whole plant | (Xie et al., 2007b; Zhu et al., 2019) |
| 333 | Other | tritriacontane | *D. cappa* | Root | (Guo et al., 2007) |
| 334 | Other | (2S,3S,4R,8E)-2-[(2'R)-2'-hydroxydocosanosylamino] -octadecane-1,3,4-triol | *D. cappa* | Root | (Guo et al., 2007) |
| 335 | Other | (2S,3S,4R,8E)-2-[(2'R)-2'-hydroxytricosanosylamino]-octadecane-1,3,4-triol | *D. cappa* | Root | (Guo et al., 2007) |
| 336 | Other | (2S,3S,4R,8E)-2-[(2'R)-2'-hydroxytetracosanosylamino] -octadecane-1,3,4 -triol | *D. cappa* | Root | (Guo et al., 2007) |
| 337 | Other | (2S,3S,4R,8E)-2-[(2'R)-2' hydroxypentacosanosylamino] -octadecane-l,3,4-triol | *D. cappa* | Root | (Guo et al., 2007) |
| 338 | Other | (2S,3S,4R,8E)-2-[(2'R)-2'-hydroxyhexacosanosylamno] -octadecane-1,3,4-triol | *D. cappa* | Root | (Guo et al., 2007) |
| 339 | Other | glicosyringicacid | *D. cappa* | Root | (Guo Qilei, 2007) |
| 340 | Other | Methyl-α-D-frucofuranoside | *D. cappa* | Root | (Guo Qilei, 2007) |
| 341 | Other | Benzyl-2-O-β-D-glucopyranosy-2，6-dihydroxybenzoate | *D. cappa* | Whole plant | (Zhou, 2017) |
| 342 | Other | benzyl alcohol glucoside | *D. cappa* | Whole plant | (Zhou, 2017) |
| 343 | Other | Juglans cerebroside A | *D. cappa* | Whole plant | (Zhou, 2017) |
| 344 | Other | 5-hydroxymethyl furfural | *D. cappa* | Root | (Lincai et al., 2017) |
| 345 | Other | 1H-indole-3-carbaldehyde | *D. nervosa* | Whole plants | (Sheng-Lan et al., 2019) |
| 346 | Other | DNP−1 | *D. pterocaula* | Root | (Wang et al., 2023) |
| 347 | Other | 3,4-Dihydroxybenzoic acid | *D. wissmanniana* | Aerial part | (wang, 2013) |
| 348 | Other | 3-Hydroxy-4-methoxybenzoic acid | *D. wissmanniana* | Aerial part | (wang, 2013) |
| 349 | Other | dibutylphthalate | *D. wissmanniana* | Aerial part | (wang, 2013) |
| 350 | Other | diisobufghctyl phthalate | *D. wissmanniana* | Aerial part | (wang, 2013) |
| 351 | Other | bis(2-ethylhexyl)-benzene-1,2-dicarboxylate | *D. pterocaula* | Whole plants | (Zhu et al., 2019) |
| 352 | Other | phthalic acid isodibutyl ester | *D. pterocaula* | Whole plants | (Sheng-Lan et al., 2019) |

**References**

Baruah, N.C., Sharma, R.P., Thyagarajan, G., Herz, W., and Govindan, S.V. (1979). New flavonoids from Inula cappa. *Phytochemistry* 18(12)**,** 2003-2006. doi: 10.1016/S0031-9422(00)82720-X.

Baruah, R.N., Sharma, R.P., Baruah, J.N., Mondeshka, D., Hertz, W., and Watanabe, K. (1982). Ineupatoriol, a thiophene analogue of ichthyothereol, from Inula eupatorioides. *Phytochemistry* 21(3)**,** 665-667. doi: 10.1016/0031-9422(82)83161-0.

Baruah, R.N., Sharma, R.P., Thyagarajan, G., Herz, W., Govindan, S.V., and Blount, J.F. (1980). Unusual germacranolides from Inula eupatorioides. *J. Org. Chem* 45(24)**,** 4838-4843. doi: 10.1021/jo01312a007.

Bohlmann, F., Ahmed, M., and Jakupovic, J. (1982). Inositol angelates from Inula cappa. *Phytochemistry* 21(3)**,** 780-782. doi: 10.1016/0031-9422(82)83188-9.

Cheng, X.-R. (2012). *Studies on bioactive secondary metabolites from four plants of inula genus.* Doctor, Shanghai Jiao Tong University

Cheng, X.-R., Wang, C.-H., Wei, P.-L., Zhang, X.-F., Zeng, Q., Yan, S.-K., et al. (2014). New sesquiterpenic acids from Inula wissmanniana. *Fitoterapia* 95**,** 139-146. doi: 10.1016/j.fitote.2014.03.013.

Cheng, X.-R., Zhang, S.-D., Wang, C.-H., Ren, J., Qin, J.-J., Tang, X., et al. (2013). Bioactive eudesmane and germacrane derivatives from Inula wissmanniana Hand.-Mazz. *Phytochemistry* 96**,** 214-222. doi: 10.1016/j.phytochem.2013.10.006.

Duoging, F., Shide, L., Huiling, W., and al, e. (1997). Studies on the Chemical Constituents of Veined Inula (Inula nervosa ). *Chinese Traditional Patent Medicine* (02)**,** 67-69. doi: 10.7501/j.issn.0253-2670.1997.2.034.

Guo, Q., Yang, J., and Liu, J. (2007). Studies on the chemical constituents from Inula cappa (II). *J. Chin. Med. Mater.* 30(1)**,** 35-37. doi: 10.13863/j.issn1001-4454.2007.01.015.

Guo Qilei, Y.J., Liu Jianxun (2007). Study on the chemical composition of Inula cappa. *Chinese traditional patent medicine* (06)**,** 887-889. doi: 10.3969/j.issn.1001-1528.2007.06.036.

Huan-yu, G., Yan-yu, L., Shang-gao, L., Jun-hong, L., Yu, H., Lin, Z., et al. (2014). Caffeoylquinic Acid Derivatives from Inula cappa. *NATURAL PRODUCT RESEARCH AND DEVELOPMENT* 26(12)**,** 1948. doi: 10.16333/j.1001-6880.2014.12.010.

LAN, Y. (2010). *Studies on the constituents from inula nervosa wall.* master Shanghai Jiao Tong University

Li, W., Yang, Y., Wu, J., Jiang, S., Yang, Y., Guo, T., et al. (2023). A new labdane diterpenoid glycoside and other constituents from Inula nervosa (Asteraceae) and their chemotaxonomic importance. *Biochem. Syst. Ecol* 109**,** 104662. doi: 10.1016/j.bse.2023.104662.

Lincai, Y., Yao, Q., Rujing, W., Xin, W., Chunmei, Y., and early, L.W. (2017). Chemical Constituent of Inula cappa DC. *Asia-Pacific Traditional Medicine* 13(06)**,** 33-34. doi: 10.11954/ytctyy.201706011.

Sheng-Lan, Z., Chun-Li, H., Yong-Zhen, X., Yang-Guo, X., Guo-Jing, W., Wei-Dong, Z., et al. (2019). Pterocaullins A− D, four sesquiterpene lactones from Inula pterocaula. *Phytochem Lett* 33**,** 70-76. doi: 10.1016/j.phytol.2019.07.010.

Tai Zhigang, C.C., Qin Benkui, Cai Le, Xu Yanqun (2014). Studies on Triterpenoid Saponins from Inula Pterocaula. *J. Kunming Univ. Sci. Technol., Nat. Sci. Ed* 39(05)**,** 70-75. doi: 10.3969 /j.issn.1007-855x.2014.05.014.

Tai Zhigang, H.X., Liu Mousheng, Yang Yaling, Qin Benkui (2013). Studies on Chemical Constituents of inula pterocaula. *Journal of Kunming University of Science and Technology ( Natural Science Edition)* 38(03)**,** 85-88. doi: 10.3969 /j.issn.1007-855x.2013.03.015.

wang, C.-h. (2013). *Studies on the constituents from inula wissmanniana and quallty control of inula cappa.* master, Shanghai Jiao Tong University

Wang, C., Zhang, X., Wei, P., Cheng, X., Ren, J., Yan, S., et al. (2013). Chemical constituents from Inula wissmanniana and their anti-inflammatory activities. *Arch. Pharmacal Res.* 36**,** 1516-1524. doi: 10.1007/s12272-013-0143-1.

Wang, F.-Y., Li, X.-Q., Sun, Q., Yao, S., Ke, C.-Q., Tang, C.-P., et al. (2012). Sesquiterpene lactones from Inula cappa. *Phytochem Lett* 5(3)**,** 639-642. doi: 10.1016/j.phytol.2012.06.012.

Wang, Z., Ma, X., Shi, S., He, S., Li, J., Wilson, G., et al. (2023). Structural Characterization and Anti-Inflammatory Activity of a Novel Polysaccharide from Duhaldea nervosa. *Polymers* 15(9)**,** 2081. doi: 10.3390/polym15092081.

Wu, J., Tang, C., Yao, S., Zhang, L., Ke, C., Feng, L., et al. (2015). Anti-inflammatory inositol derivatives from the whole plant of Inula cappa. *J. Nat. Prod* 78(10)**,** 2332-2338. doi: 10.1021/acs.jnatprod.5b00135.

Wu, Y., Yang, Y., Dong, M., Sauriol, F., Shi, Q., Gu, Y., et al. (2014). A new taraxastane-type triterpene from the flowers of Inula cappa. *Chemistry of natural compounds* 50**,** 850-852. doi: 10.1007/s10600-014-1098-2.

Wu, Z.-J., Shan, L., Lu, M., Shen, Y.-H., Tang, J., and Zhang, W.-D. (2010). Chemical constituents from Inula cappa. *Chemistry of natural compounds* 46**,** 298-300. doi: 10.1007/s10600-010-9595-4.

Wu, Z., Shen, Y., and Zhang, W. (2013). Two new phenolic glycosides from Inula cappa DC. *Natural Product Research* 27(8)**,** 719-722. doi: 10.1080/14786419.2012.691496.

Xie, H.-G., Chen, H., Cao, B., Zhang, H.-W., and Zou, Z.-M. (2007a). Cytotoxic germacranolide sesquiterpene from Inula cappa. *Chem Pharm Bull* 55(8)**,** 1258-1260. doi: 10.1248/cpb.55.1258.

Xie, H., Zhang, H., Zhang, J., Xu, L., and Zou, Z. (2007b). Chemical constituents from Inula cappa. *Chin. J. Nat. Med* 5(3)**,** 193-196. doi: 672-3651（2007）03-0193-04.

Xie Huiting, W.J., Chen Zhendong, Yuan Gaoqing, Li Qi, Qin Linwei (2012). Antimicrobial Compound Isolated from Inula cappa and Its Inhibitory Characteristics. *Nat Prod Res Dev* 24(11)**,** 1534-1537. doi: 10.16333/j.1001-6880.2012.11.021.

Yan, L., Cheng, X.R., Zeng, Q., Qin, J.J., Zhang, W.D., and Jin, H.Z. (2011). Phytane and neoclerodane diterpenes from the aerial parts of Inula nervosa Wall. *Biochem. Syst. Ecol* 39(4-6)**,** 700-703. doi: 10.1016/j.bse.2011.06.001.

Yan, L., Huang, Y., Fu, J.J., Qin, J.J., Zeng, Q., Zhu, Y., et al. (2010). Three New Phenylpropanoids from Inula nervosa Wall. *Helv Chim Acta* 93(7)**,** 1418-1421. doi: 10.1002/hlca.200900432.

Yan LAN Jin Huizi, N.L., Qin Jiang, Fu Jianjun, Zhang Weidong (2011). Chemical Constituents from Inula nervosa W all. *Ｎat Prod Res Dev* 23(02)**,** 258-261. doi: 10.16333/j.1001-6880.2011.02.014.

Yang, Y., Wang, Y., Zhao, L., Dong, M., Huo, C., Gu, Y., et al. (2011). Chemical constituents of Inula cappa flowers. *Chin Tradit Herbal Drugs* 42**,** 1083-1086. doi: CNKI:SUN:ZCYO.0.2011-06-012.

Zheng, L., Hao, X., Yuan, C., Huang, L., Zhang, J., Dong, F., et al. (2015). Study on chemical constituents of Inula cappa. *China J Chin Mater Med* 40(4)**,** 672-678. doi: DOI: 10.4268/cjcmm20150419.

Zhou, W. (2017). Chemical constituents of Inula cappa. *J Chin Pharm Sci***,** 25-30. doi: 10.11669/cpj.2017.01.005.

Zhu, S., Xiao, Y., Wu, G., Xie, Y., Muhammad, I., Sun, Y., et al. (2019). Chemical constituents of Inula pterocaula. *Chemistry of Natural Compounds* 55**,** 1135-1137. doi: 10.1007/s10600-019-02913-2.

Zou, Z.-M., Xie, H.-G., Zhang, H.-W., and Xu, L.-Z. (2008). Inositol angelates from the whole herb of Inula cappa. *Fitoterapia* 79(5)**,** 393-394. doi: 10.1016/j.fitote.2007.11.031.
